# Multi-path direct current spinal stimulation extended survival in the SOD1-G93A model of amyotrophic lateral sclerosis

**DOI:** 10.3389/fneur.2025.1594169

**Published:** 2025-06-26

**Authors:** Zaghloul Ahmed, Sreyashi Samaddar, May Hassieb, Rodina Sadek, Viktoriya Morozova, Sultana Begum

**Affiliations:** ^1^Department of Physical Therapy, The College of Staten Island, City University of New York, Staten Island, NY, United States; ^2^Center for Developmental Neuroscience, The College of Staten Island, City University of New York, Staten Island, NY, United States; ^3^Graduate Center, City University of New York, New York, NY, United States; ^4^Department of Biology, Brooklyn College, City University of New York, Brooklyn, NY, United States; ^5^Helene Fuld College of Nursing, New York, NY, United States

**Keywords:** ALS (amyotrophic lateral sclerosis), direct current stimulation (tDCS), spinal motor neuron, survival, neuroprotection

## Abstract

**Introduction:**

Amyotrophic lateral sclerosis (ALS) is a progressive neurodegenerative disease that affects motor neurons in the spinal cord and brain. We have developed a novel non-invasive approach, MultiPath-DCS, which utilizes direct current stimulation at multiple sites along the neural axis to provide simultaneous spinal and peripheral stimulation targeted at the affected limbs. MultiPath-DCS modulates the excitability of spinal cord neurons. This effect is significant for ALS, as motor neuron hyperexcitability is a fundamental characteristic of the disease.

**Methods:**

This study used a transgenic mouse model of ALS (SOD1-G93A). Anodal-MultiPath-DCS was applied with six electrodes: three on the spine (centered on T13 and with an anodal polarity), two on the sciatic nerves (one on each nerve), and one on the abdomen. Mice were divided into two groups (stimulated vs. unstimulated or sham-stimulated). The stimulated animals received stimulation for one hour a day, three times a week, for three weeks. Survival was calculated from the onset of the disease and birth until the animal’s endpoint. We also performed various electrophysiological and molecular experiments to uncover the mechanism of action.

**Results:**

We demonstrated molecular changes induced by anodal MultiPath-DCS, including (a) reduced expression of mutant SOD1 protein, (b) decreased expression of elevated NKCC1, (c) reduced phosphorylated tau, (d) increased expression of HSP70, and (e) increased expression of LC3B. Additionally, we found that treatment with Anodal-MultiPath-DCS (anode on the spinal column) reduces long-term neuronal spinal excitability, slows the progression of muscle weakness, and extends the lifespan of stimulated mice. The mean survival time in the control group was 12.4 days. In comparison, the mean survival time in the stimulated group was 21.6 days using a therapeutic stimulation paradigm, representing a 74% increase in survival from disease onset. Spinal motor neuron survival showed a 54% increase in stimulated compared to non-stimulated groups.

**Discussion:**

Combined, this data provides evidence that Anodal-MultiPath-DCS reduces hyperexcitability and enhances the clearance of misfolded proteins by modulating autophagy and proteolytic systems. By decreasing spinal excitability and clearing toxic proteins from motor neurons, Anodal-MultiPath-DCS promotes survival and could serve as a disease-modifying intervention for ALS.

## Introduction

Amyotrophic lateral sclerosis (ALS) is a progressive neurodegenerative disease that affects motor neurons in the spinal cord and brain. ALS causes muscle weakness, paralysis, and eventually death. Someone is diagnosed with – or dies from – ALS every 90 minutes (https://www.als.org/). According to the ALS Association, nearly 5,000 people are diagnosed with ALS yearly, and at least 16,000 live with the disease at any given time in the US alone. There are 3 FDA-approved drugs for ALS (FDA-Approved Drugs for Treating ALS | The ALS Association). However, the effectiveness of these drugs is limited, and they do not substantially increase survival in patients with the disease. Therefore, investigating new potential approaches to slowing ALS disease progression, especially through bioelectronics that can non-invasively modulate the activity of spinal motor neurons, is urgently needed due to its excellent safety, growing evidence of its relevant modulatory effects on biological pathways, and ability to quickly and efficiently be brought into clinical use.

SOD1-ALS is one of the most aggressive forms of familiar ALS that is found in approximately 20% of familial forms of ALS and about 5% of the sporadic forms of ALS (depending on the demographic) (1). SOD1 is an intrinsic scavenger of superoxide anions that protects cells from damage by radical oxygen species. The mutated form of superoxide dismutase promotes misfolding and aggregation of the protein in the motor neurons in the spinal cord, brainstem, and brain. It has also been reported that mutated SOD1 disrupts glutamate homeostasis and therefore leads to AMPA and NMDA-mediated glutamate excitotoxicity (2). The current study investigates the effects of a novel form of multi-site neuromodulation, which we refer to as Anodal-MultiPath-DCS, on the SOD1 mouse model of ALS. The anodal term refers to the placement of the anodal side of the circuit on the dorsum of the spinal column.

Spinal direct current stimulation (DCS) modulates electrophysiological characteristics of spinal cord activity depending on polarity (anode vs. cathode), the direction of current relative to neuronal orientation, current density, duration, and the number of repetitions of stimulation. A combination of these parameters can increase or decrease spinal cord (or cortical) neuronal excitability. This ability of DCS to modulate excitability is relevant to ALS as neuronal hyperexcitability is a known factor of ALS pathology (9, 64–67, 38). Spinal hyperexcitability could be reduced by DCS (8, 23–26, 68), thereby increasing the likelihood of reducing the progression of disease in ALS patients. DCS also improves local blood flow in stimulated spinal cord tissue (50). A reduction in blood flow is associated with neuronal degeneration (69). Moreover, we have demonstrated that DCS enhances the number of newly born neural cells in the spinal cord and upregulates growth factors, including BDNF (50) and VEGF (unpublished). Interestingly, treatment of the spinal cord with anodal DCS was found to downregulate NGF, which is known to be overexpressed in ALS patients and was proposed to be produced through an inflammatory process that activates astrocytes. Astrocytic NGF activates the re-expressed p75NTR receptor on motor neurons and leads to cell death (2). We have also reported that DCS modulates excitatory factors such as the expression of the sodium-potassium-chloride cotransporter 1 (NKCC1) (6), glutamate release, calcium levels (70), and Na-K ATPase pump expression and activity (3) which was found impaired in patients with ALS (45). In addition, DCS upregulates heat shock proteins HSP70 and might activate autophagy and the proteasomal degradation pathways (6). Taken together, DCS modulates physiological and molecular mechanisms in manners that could directly or indirectly impact motor neuron survival in ALS.

Non-invasive electrical and magnetic brain stimulation studies have shown the potential to slow disease progression in ALS patients (4, 5). However, studies investigating whether spinal DCS can slow the progression of ALS have been limited. The current study aims to test the effects of therapeutic intervention on the progression of motor dysfunction and survival rate. Transgenic SOD1 mice were stimulated with Anodal-MultiPath-DCS (A-MultiPath-DCS) starting from disease onset. We hypothesized that A-MultiPath-DCS starting at disease onset would reduce spinal hyperexcitability and upregulate beneficial molecular mechanisms that could slow the progression of the disease.

## Methods and materials

### Ethical approval

All animal experiments conformed to the Public Health Service (PHS) policy on humane care and the use of laboratory animals. The experimental procedure of the current study was reviewed and approved by the College of Staten Island Institutional Animal Care and Use Committee (IACUC), Protocol Number 13-017.

### Animals

Animals were bred in our animal facility. Male B6SJL-Tg(SOD1*G93A)1Gur/J, Genotype: Hemizygous, were acquired from the Jackson Laboratory (Bar Harbor, ME, USA) and used to start the colony. Offspring were genotyped and randomly assigned into the stimulated SOD1 carrier (SOD1-S) or nonstimulated/sham SOD1 carrier control (SOD1-NS) groups. Transgene copy numbers were evaluated using real-time quantitative PCR by determining the difference in threshold cycle (ΔCT) between the transgene (hSOD1) and a reference gene. Mice in treated and untreated groups were matched in sex and ΔCT numbers. We also included a wild-type control group (WT) for behavioral, histological, and electrophysiological comparisons. The animals were tagged by removing one of the toes, and stimulated and non-stimulated control animals were housed in the same cage as an extra environmental control. Animals were given a number, and cages were numbered and color-tagged for experimental blindness. The total number of animals used in this project was 73 mice (39 males and 34 females) divided equally among the treatment groups (sham or non-stimulated vs. stimulated). For the survival experiments, 49 mice were used (SOD1-S, *n* = 23; SOD1-NS, *n* = 25). To test motor evoked potentials (MEP), 17 animals were used. Seven animals were used for the motor neuron count experiment. Another set of animals was used for the prophylactic experiments (*n* = 45; see Supplementary material) DOI 10.5281/zenodo.13955488. In all groups, animals were matched in age, copy number, female vs. male, and disease stage. Animals were fed ad-lib PurinaMouse Chow (LabDiet, St Louis, MO, USA), and at later stages of the disease, the feed was wetted/softened and then added to the cages to assist feeding. Animals housing followed 12 h of light/dark cycle. Room temperatures were kept at 22°C.

### Behavioral assessments

**Figure 1:**
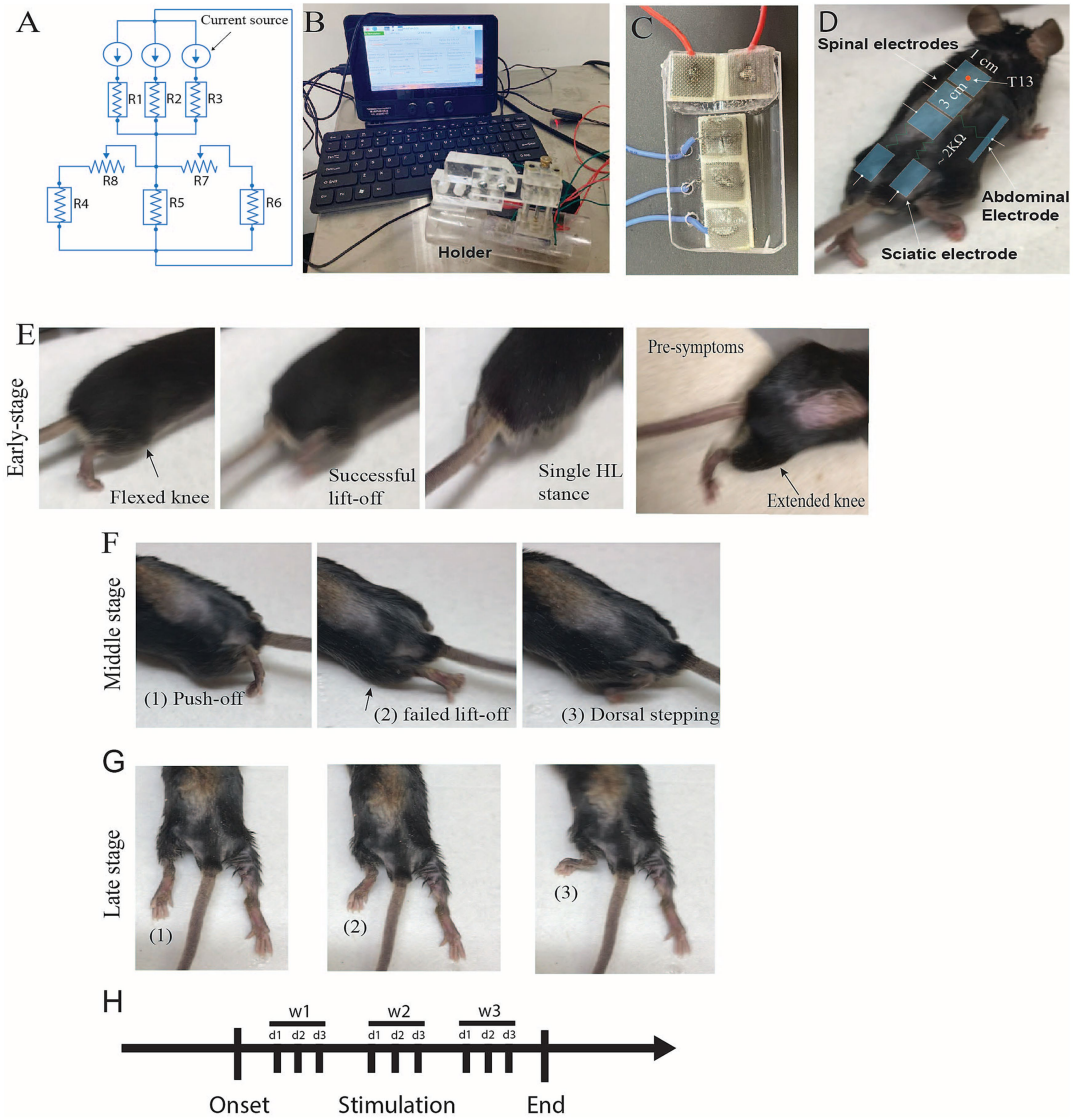
Stimulation circuit, device, mouse setup, and stimulation paradigm. **(A)** Equivalent circuit; R1, R2, and R3 are spinal electrodes (electrode-skin interface resistance); R4 and R6 are sciatic electrodes; R5 is the abdominal electrode; R7 and R8 are variable resistances that adjust the current passing through R4, R5, and R6. **(B)** The MultiPath DCS device was designed and manufactured by PathMaker Neurosystems Inc., incorporating the circuit shown. **(C)** Spinal and sciatic electrodes are assembled, displaying the three spinal and two sciatic electrodes (top). **(D)** Drawings on a SOD-1 mouse depict the location and sizes of electrodes. T13 is the thirteenth vertebra used as an electrode placement marker. **(E–G)** Stages of motor dysfunction in SOD-1 model mice. Serial images were taken from a video recording of the SOD-1 mouse. **(E)** Ground walking is in the early stage; the knee is not flexed during stepping, and there is an abnormal pattern in lift-off and single stance (but successful). Right: an image from a mouse in a pre-symptomatic stage contrasts with the stages of the disease. Left. **(F)** Ground walking during the middle stage. In this stage, push-off is partially successful, but lift-off fails and occasional dorsal stepping occurs. The arrow in E also indicates the mouse using the knees as a brace. **(G)** Late-stage images show slight HL movement (1–3). Note that the right leg is extended with no movement. **(H)** The timeline of the survival experiment shows onset, stimulation frequency (1 h per day, three times a week), and conclusion.

Walking on a grid was performed to test the animals’ hindlimb motor function. The test was described previously (6). We used a six-point foot fault scoring system that goes from zero (complete miss) up to 6 points (correct placement on the rungs of the grid). Videos were taken from the underside of the animals to reveal the placements of the hind paws of the animal on the rungs. Videos were then uploaded on a computer and analyzed by an experimenter/s who was blinded to the animals’ treatment condition. To capture the onset of the disease and its progression, assessments were performed every week after the animals reached the age of 60 days, and then the frequency of the evaluations was accelerated to every day after the age of 90 days. Animals were considered to reach the onset of the disease if their grid walking score reached 80% of the maximal score of 100% (normal score). In addition to the above criteria, animals’ trunk posture, balance, tail use and posture, and muscle wasting and tremor of the hind limb were monitored, see Table 1. Based on the above, animals’ progression was divided into 4 stages (early, middle, late, and terminal stages), see Table 1. Disease onset, the early stage, is considered to begin when the animal shows body shaking, paw slippage (80% reduction or a score of 4 in the grid test), tail down, slow locomotor pattern, and longer delay before initiating locomotion when walking on a metal grid. Two other stages, middle and late, were also identified, see Table 1. These functional stages are used to track the progression of the disease and determine the survival period in response to treatment. A picture profile of an example animal at different stages of progression is shown in Figure 1. These stages do not have equal rates of disease progression. For example, later stages progress much quicker than early stages. The animal takes a few days to progress from the late stage to the terminal endpoint, which is when the animal cannot right itself from a supine position within 20 s. The motor signs of different stages shown in Table 1 occur concurrently. We observed that the motor signs progress from one stage to the next in an abrupt manner and in a short period that is as short as overnight. A trained, blinded investigator performed daily monitoring of the signs and symptoms of the disease in all animals.

**Table 1:** Determination of the functional level of SOD1-G93A mice.

Stages of the disease progression
Early stage: The individual exhibits plantar stepping with incomplete weight support and slightly flexed knees Trunk instability is observed as a wobble at the base of the tail There is noticeable paw slippage, a slow locomotor pattern, and a longer delay before initiating movement when walking on a metal grid with dimensions of 2.5 × 2.5 cm. While walking on the grid, the tail is positioned downwards and wrapped around the rungs for added stability. A tremor is present in the toes of the hindlimbs, but not throughout the entire limb.
Middle stage: The plantar placement of the paw without weight support Inability to lift the body at the base of the tail Failure to extend the knees to lift the body during stepping Using the knees to brace the body while stepping (Figure 1B) Occasional, frequent, or consistent dorsal stepping A tremor in the toes and the entire hindlimb
Late stage: No or slight ankle movement Extended hindlimb position due to spasm Muscle wasting in the hindquarters of the body Able to drag itself forward using forelimbs Able to right itself in less than 20 seconds Tremor
Terminal end-point: Generalized muscle wasting (fore and hind limbs and trunk) Inability to pull itself forward Severe trunk kyphosis Inability to right itself in less than 20 seconds

### MultiPath-DCS apparatus

A 4-channel DC stimulator (MultiPath-DCS device) that was manufactured specifically for this project by PathMaker Neurosystems Inc. (Boston, MA) and funded by an NIH STTR grant (#R41NS125872) was utilized in the described studies. The MultiPath-DCS device is a research device for animal studies providing 3 channels of simultaneous Direct Current Stimulation to surface electrodes. Each channel consists of a “high side” current source and a “low side” current sink. All current sources and sinks can be independently set and controlled. Each high side can source up to 3 mA constant current with > 85 V compliance voltage. Low-side current sinks limit and control the return currents. The high-side source current is typically connected to a stimulation anode, and the low-side sink current is typically connected to a stimulation cathode. This arrangement allows the current flow to be “steered” among electrodes for optimum therapy delivery to the test subject. A circuit diagram is shown in Figure 1A, and the MultiPath-DCS device is shown in Figure 1B. All stimulation currents can be independently set and controlled. Parameter setting is accomplished through slider controls on either the touch screen directly or a three-button mouse. Additionally, knobs are provided for setting currents for Channels 1–3. Current through each electrode is independently measured and displayed in real-time. The device offers a stimulation session countdown timer and terminates the stimulation delivery at the end of the preset time of up to 6 h. A single electrode cable interfaces from the device to the subject animal. A single hardware button terminates all therapy instantly. We designed a mouse holder fixed with electrodes, as shown in Figures 1B,C. The stimulator has 3 current sources and was connected, as shown in the circuit in Figure 1A. The spinal electrode was constructed of 3 smaller electrodes to achieve uniformity in the current distribution in the spinal column and cord. The primary rationale for using 3 electrodes on the spine is to reduce the electrode size to ensure uniformity of the current distribution. Also, two electrodes are placed on each sciatic region, as shown in Figure 1C. The reference electrode was placed on the abdominal region. The current intensity in each spinal and sciatic electrode was set at 0.55 mA. The abdominal electrode was made larger to act as a sink for the total current; spinal electrodes were connected to the anode stimulation sources, and sciatic and abdominal electrodes were connected to cathodal stimulation sources. Specifically, the most rostral spinal electrode (at T13) was the anode, and its reference cathode electrode was the abdominal electrode. The reference cathode electrode for the middle spinal anode electrode was placed on the right sciatic region. The most caudal anode spinal electrode was connected to the left sciatic region cathode electrode. The rationale for the sciatic nerve spinal cord arrangement is to steer the current toward the ventral part of the spinal cord and through the initial segment of the spinal nerves. This arrangement was more effective in suppressing the spinal motor neuron output. In the mouse holder, the electrodes were made of stainless-steel mesh covered by a hydrogel in contact with the animal’s skin (Figure 1C). The hydrogel was changed after every session. The skin under the electrodes was shaved and wetted with 3% saline before stimulation. When the animals were placed in the holder, the hind limbs were locked into two openings that prevented the animals from changing the location of the electrodes on the spine. The sciatic electrodes were also raised slightly higher to fit the contours of the mice’s bodies. The holder was made adjustable to allow precise fitting for different animal sizes, providing accurate placement for the electrodes. Following the determination of the onset of disease, animals were stimulated 3 times a week for 60 min per day. The skin was cleaned with alcohol after each session; the investigators also monitored any injuries and treated them immediately with an antibiotic and cleaned them with antiseptic solutions as needed.

### Immunohistochemistry (IHC)

Animals were anesthetized with IP injections of ketamine and xylazine 90 and 10 MG/kg, respectively. Animals were perfused with cold 0.9% saline followed by 4% paraformaldehyde (PFA). The spinal column was then removed and incubated in 4% PFA overnight. On the second day, the spinal cord was removed from the spinal column and placed in 30% sucrose for another 24 h. The spinal cords were then sectioned using a cryostat into 30um slices. The staining intensity was measured in motor neurons from at least 2 slices per animal from the thoracolumbar segment of the spinal cord.

#### ChAT and NKCC1 IHC

Spinal cord sections were washed three times in phosphate buffer saline (PBS). The sections were incubated in 10% Rabbit serum in 0.1% Triton X-100 in 0.1 M PBS for 1 h at room temperature. Then, the sections were incubated with Anti-Choline Acetyltransferase Antibody (ChAT) primary antibody (AB144P, EMD Millipore Corp. Burlington MA; 1:500) in 2% rabbit serum in 0.1% Triton X-100 in 0.1 M PBS solution for 48–72 h at 4°C. Then, sections were washed three times in PBS, 10–15 min each. Then, sections were incubated with Rabbit Anti-Goat 568 nm (Molecular Probes; 1:500) in 2% rabbit serum in 0.1% Triton X-100 in 0.1 M PBS solution for 1 h at room temperature. Sections were again washed three times in 0.1 M PBS and blocked with 10% goat serum in 0.2% Triton X-100 in 0.1 M PBS for 1 h at room temperature, and then followed by overnight incubation with Na-K-Cl Cotransporter (NKCC1) primary antibody (sc: 514774, Santa Cruz Biotechnology Dallas, TX; 1:400) in 2% goat serum in 0.2% Triton X-100 in 0.1 M PBS solution at 4°C. Then, sections were washed three times with 0.1 M PBS and incubated with Goat anti-Mouse 488 (Invitrogen; 1:500) in 2% goat serum in 0.2% Triton X-100 in 0.1 M PBS solution for 1 h at room temperature. Sections were washed thrice with 0.1 M PBS, mounted on glass slides, and covered with glass coverslips using a mounting medium with DAPI (H-1200; Vector Laboratories Inc). *ChAT* immunostaining was also used to mark surviving motor neurons to investigate the effect of A-MultiPath-DCS on motor neuron degeneration in SOD1 mice.

#### HSP70 IHC

Primary mouse monoclonal HSP70 (F-3) antibody was added (1:500, sc-373867, Santa Cruz Biotechnology), and the sections were incubated overnight on a shaker at 4°C. The next day, the sections were washed 3x and then incubated with a biotinylated goat anti-mouse antibody (1:500; BA-9200, Vector Laboratories Burlingame, CA USA). After 1 h, DyLight 488 (1:2000; SA-5488, Vector Laboratories) was added. After another 30 min, the sections were washed 2x with Tris A before the slices were mounted with medium containing DAPI (H-1200; Vector Laboratories).

#### h-SOD-1 IHC

Segments of the spinal cord underneath the injury site were cryo-sectioned to prepare multiple 30 μm sections. The sections were washed with 0.1 M PBS buffer 2 times (10 min each) to remove the extra TissueTek medium. The sections were subjected to antigen retrieval by treating them with 10 mM sodium citrate, pH:6 at 95°C for 5–10 min in a water bath. Sections were again rinsed twice, followed by incubation with a blocking buffer of 10% normal goat serum (NGS) in 0.3% Triton X-100 in 0.1 M PBS for at least 1 h at room temperature. After an hour, the sections were incubated with primary antibody for SOD-1, mouse monoclonal antibody (1:400) (sc-101523; Santa Cruz Biotechnology, Dallas, TX, USA) in 2% NGS and 0.3% Triton X-100 in 0.1 M PBS and left overnight at 4°C. The next day, the sections were rinsed with 0.1 M PBS buffer 3 times (10–15 min each) and incubated with secondary antibody Alexa Fluor 488 goat anti-mouse IgG (1:500) (A 11001: Invitrogen; Carlsbad, CA, USA) against the SOD1 in 2% NGS and 0.1% Triton X-100 in 0.1 M PBS for 1 h. The sections were again rinsed with 0.1 M PBS buffer 3 times (10–15 min each) to wash away extra antibody solution and then incubated with DAPI (1: 5000) in 0.1 M PBS buffer for 20 min at Room temperature. The sections were again rinsed with 0.1 M PBS buffer 3 times (10–15 min each) and then mounted on glass slides using coverslips with Vectashield Mounting Medium (H-1200; Vector Laboratories; Burlingame, CA, USA). Staining intensity was measured in the same light setup conditions. Immunostained sections were imaged with a fluorescence microscope (Microscope Axio 214 Imager. A2, Carl Zeiss Microscopy, LLC, USA) and with a confocal microscope (Leica 215 Microsystems, Heidelberg, Germany).

### Electrophysiological and stretch reflex studies

To study the excitability of the spinal cord over time in transgenic mice, we used a stretch device that was published previously (6) to measure the stretch reflex in the triceps surae muscles. These measurements were performed in a similar order as the grid walking assessment. The animals were placed in an adjustable mouse holder that was designed to fix one of the hind limbs in a stable position to allow the device to stretch the target muscles in a reproducible manner; see our previous publication for more details (6). The device used a force sensor which was connected serially to the motor to measure the resistance in the muscle tissue during a stretch. Needle electromyography (EMG) from the target muscles was also simultaneously recorded. The initial position of the ankle joint was set at a 90-degree range relative to the leg. The amplitude of the stretch was 4 mm of linear displacement or 20 degrees from the 90-degree ankle position. It should be noted that all stretches were done with the same settings at all times and in all groups. Stretch reflexes (EMG and force) were collected before and after the onset of the disease and analyzed. Stretch-induced EMG was collected as root mean square EMG (RMS EMG) area and amplitude. The root mean square was calculated using a LabChart built-in equation with a window of 0.1 s that was unified among all recordings. We also collected tremor-associated EMG activity (TA-EMG) from the muscles at all stages of the disease. This EMG activity was in the form of single-unit recordings, which allowed measurements of the firing rate of the active units. Using spike analysis software, these units could be isolated and analyzed separately. The frequency rate was presented as the average of all units recorded for each session and then compared across conditions. To prove that the activity has a spinal or supraspinal origin, animals were briefly anesthetized with isoflurane, which completely abolished TA-EMG that readily returned once isoflurane was removed. Isoflurane was shown to have no direct effect on muscles or neuromuscular junction (7), which indicates that the site evoking TA-EMG is located centrally, most likely in the spinal cord. Recordings were passed through a head stage, amplified using the modified model 1700 differential AC Amplifier (A-M systems), and digitized at 10 kHz. A NeuroAmp amplifier and Ex-Head stage were used to record single units. The signal was digitized at 10 kHz. A PowerLab data-acquisition system and LabChart 8 software (ADInstruments) with spike histogram and peak analysis modules were used to acquire and analyze the data.

### Measuring motor evoked potentials (MEP)

For this particular experiment, a separate group of mice was used. Mice were at the same age from the same litter (Control non-carrier: *n* = 5; carrier non-stimulated: *n* = 7; carrier stimulated: *n* = 5). Animals were stimulated with A-MultiPath-DCS 5 times over 10 days period following the onset of disease (onset was on average at the age of 113 ± 9.8 days). Following 10 days, animals were anesthetized with ketamine and xylazine 90 and 10 MG/kg, respectively. Two small craniotomies were performed to expose the left and right cortices of the left and right hindlimb muscle representations (8). Stimulation and recording protocol started 20–30 min after injection of the anesthesia to allow recovery of the nervous tissues. A concentric needle-stimulating electrode was used to evoke MEP. This electrode was chosen for its accuracy in stimulating a localized area of the cortex. The EMG was recorded from the contralateral triceps surae muscles using needle electrodes. The active and reference recording needle electrodes were separated by about 0.5 mm, and the ground electrode was a clip that was attached to an exposed skin area. Cortical stimulation parameters were made at one pulse and 6 pulses, stimulus duration was 1 ms, and train frequency was 333 Hz. Testing was performed at 0.5, 1, 1.5, 2 and 3 mA stimulus intensities. To test for the threshold, stimulus intensities were gradually increased from 0.1 mA up to the threshold, which is defined as the stimulus intensity at which MEP was first evoked. MEP responses were converted using the common root mean square (RMS) formula. MEP amplitude, number of oscillations, and threshold were collected and used to analyze the difference between stimulated and non-stimulated carrier.

### Tremor measurements

Tremors were recorded using a miniature goniometer MLTS720 (ADInstruments). This is a flexible joint angle sensor that was used to measure changes in the angle of the ankle joint of the hind paws. The sensor was connected directly to the PowerLab Pod port for recording. The sensor end was mounted on the paw with flexible adhesive tape. The output of the goniometer was recorded on a different channel simultaneously with EMG on LabChart. A-MultiPath-DCS intensity gradually increased from 0.5 mA up to 1.7 mA. At a range of A-MultiPath-DCS intensities (0.5 to 1.5 mA), tremors mostly disappeared. Higher intensities (>1.5 mA) cause the reappearance of tremors and intensify them. Therefore, in 5 animals, we used the A-MultiPath-DCS intensity of 1.5 mA to suppress tremors. Concurrent video recording was performed to monitor tremors visually. Two parameters were used to quantify tremors: (1) the amplitude measured as the signal level relative to baseline; (2) the tremor period as the time interval (seconds) between two peaks.

### Motor neuron survival

In this experiment, mice from the same litter were randomly assigned to either SOD1-S or SOD1-NS. Three groups of mice were used to investigate spinal motor neuron survival: Wild-type (*n* = 4), SOD1 non-stimulated (NS, *n* = 6), and SOD1 stimulated (S, *n* = 5). SOD1 mice started real or sham intervention at the onset of the disease. At the onset, mice in the SOD1-S group were stimulated every other day for 5 days. All animals were sacrificed 10 days from the day of disease onset. In all animals, the upper lumber spinal cord region was sliced (2 to 3 slices/mouse) and immunostained for ChAT to mark and count motor neurons. High-magnification images of the ventral horns were taken using fluorescence and confocal microscopy. DAPI staining was also used to identify motor neuron nuclei. A Z-stack was also used to ensure accuracy in the identification process of the motor neuron. A counter function in Adobe Photoshop was used to count the neurons. In all IHC experiments, the imager and analyzer were blinded to the experimental conditions of the animals.

### Statistical analysis

Survival rate was calculated from two points of time: (1) the day of disease onset; and (2) the date of birth. The data were analyzed using Kaplan–Meier (K-M) analysis to calculate survival among stimulated and non-stimulated groups. Breslow (generalized Wilcoxon) was used to test the equality of survival distribution among the groups. An independent t-test was also used to compare the means of survival time among the different groups. This was added because of its frequent clinical use and to facilitate comparison to other studies. Comparison was made between survival days calculated from the day of onset. Survival was also compared from the day of birth to the day of death. A paired t-test (before vs. after scenarios) was used to compare electrophysiological and stretch reflex results. A repeated measures ANOVA was used when comparing TA-EMG at different stages of the disease (pre-symptomatic, early stage, late stage, and after treatment) and in comparing the effect of A-MultiPath-DCS on tremor, MEP, and NKCC1 expression. ANOVA followed by multiple comparisons *post hoc* tests were used to compare the number of motor neurons between SOD1-S, SOD1-NS, and wild type. SPSS software was used to perform all of the statistical tests (IBM StatistiSOD1-S, SPSS version 27). To test for sphericity, Mauchly’s test in SPSS was applied (*p* > 0.05). The critical level of significance was set at *p* < 0.05. Data are presented as the mean ± standard deviation in the text or 95% intervals in the figures.

## Results

### Anodal multi-path DC stimulation increased the survival time of mSOD1^G93A^ mice

**Figure 2:**
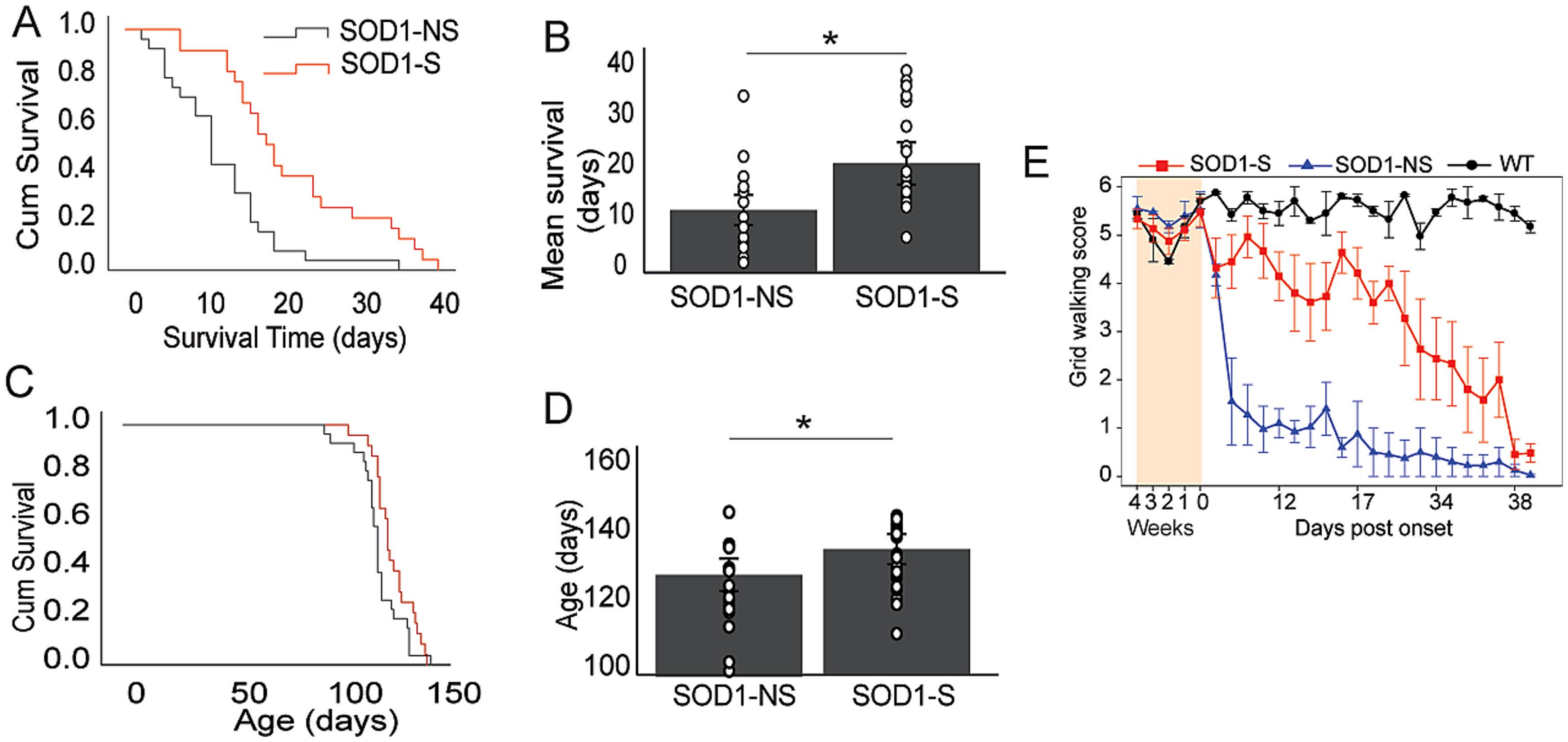
A-MultiPath-DCS increased the survival time and slowed down motor dysfunction in SOD1 mice. **(A,B)** Survival time was calculated from the day of disease onset. K-M analysis shows that therapeutic stimulation with A-MultiPath-DCS significantly increased the survival time of the transgenic mice. The stimulated group (*n* = 23) had a significantly longer survival time compared to the unstimulated group (*n* = 25), as tested by Breslow (generalized Wilcoxon): Chi-Square = 12.25, *p* = 0.0005. We also conducted a comparison between the unstimulated and stimulated groups using an independent t-test, which showed a significant difference (*p* = 0.023). **(C,D)** The survival time was calculated from the date of birth of the animals. K-M analysis showed a significant difference in the survival time, as tested by Breslow (generalized Wilcoxon) (*p* = 0.018), **(C)** we also conducted a comparison between the unstimulated and stimulated groups using an independent t-test, revealing a significant difference (*p* = 0.031). **(D)** Overall, the data demonstrate that therapeutic A-MultiPath-DCS stimulation prolonged animal survival time when survival is calculated either from disease onset or from birth to death. **(E)** A line diagram shows average changes in motor function in a subgroup of animals (tested by the walking grid test). The shaded area represents scores before the onset of the disease. SOD1-NS, non-stimulated SOD1 carrier mice; SOD1-S, stimulated SOD1 carrier mice; WT, wild-type mice.

First, we calculated the survival time from the day of disease onset. K-M analysis shows that therapeutic stimulation with A-MultiPath-DCS significantly increased the survival time of the transgenic mice. As shown in Figures 2A,B, the stimulated SOD1 group has a significantly longer survival time (21.6 ± 2.0 days, *n* = 23) compared to the unstimulated SOD1 group (12.4 ± 1.4 days, *n* = 25), a difference of 9.2 days. The statistical significance was tested using Breslow (generalized Wilcoxon): Chi-Square = 12.25, *p* = 0.0005. We also compared unstimulated and stimulated groups using an independent t-test, showing a significant difference (*p* = 0.023) (Figure 2B). There is about 9 days or a 74% increase in the survival time for the stimulated group.

We also calculated the survival time from birth for control and stimulated animals. As shown in Figures 2C,D, K-M analysis showed a significant difference in the survival time as was tested by Breslow (generalized Wilcoxon) (*p* = 0.018). The mean age at death for the stimulated group was 136.6 ± 2.1, and for the non-stimulated group was 129.7 ± 2.3. A difference of 7 days was statistically significant, independent t-test (*p* = 0.031) (Figure 2D). The data shows that therapeutic A-MultiPath-DCS stimulation prolonged animal survival time when survival is calculated from disease onset or birth to death.

In a sub-group of animals (control, *n* = 4; stimulated, *n* = 4), we plotted the grid walking scores as shown in Figure 2E. The line graph shows slower progression of motor dysfunction in treated animals, which indicates that treatment not only leads to longer survival time but preserves motor function as well.

### Anodal multi-path DC stimulation reduced spinal excitability

#### TA-EMG activity

**Figure 3:**
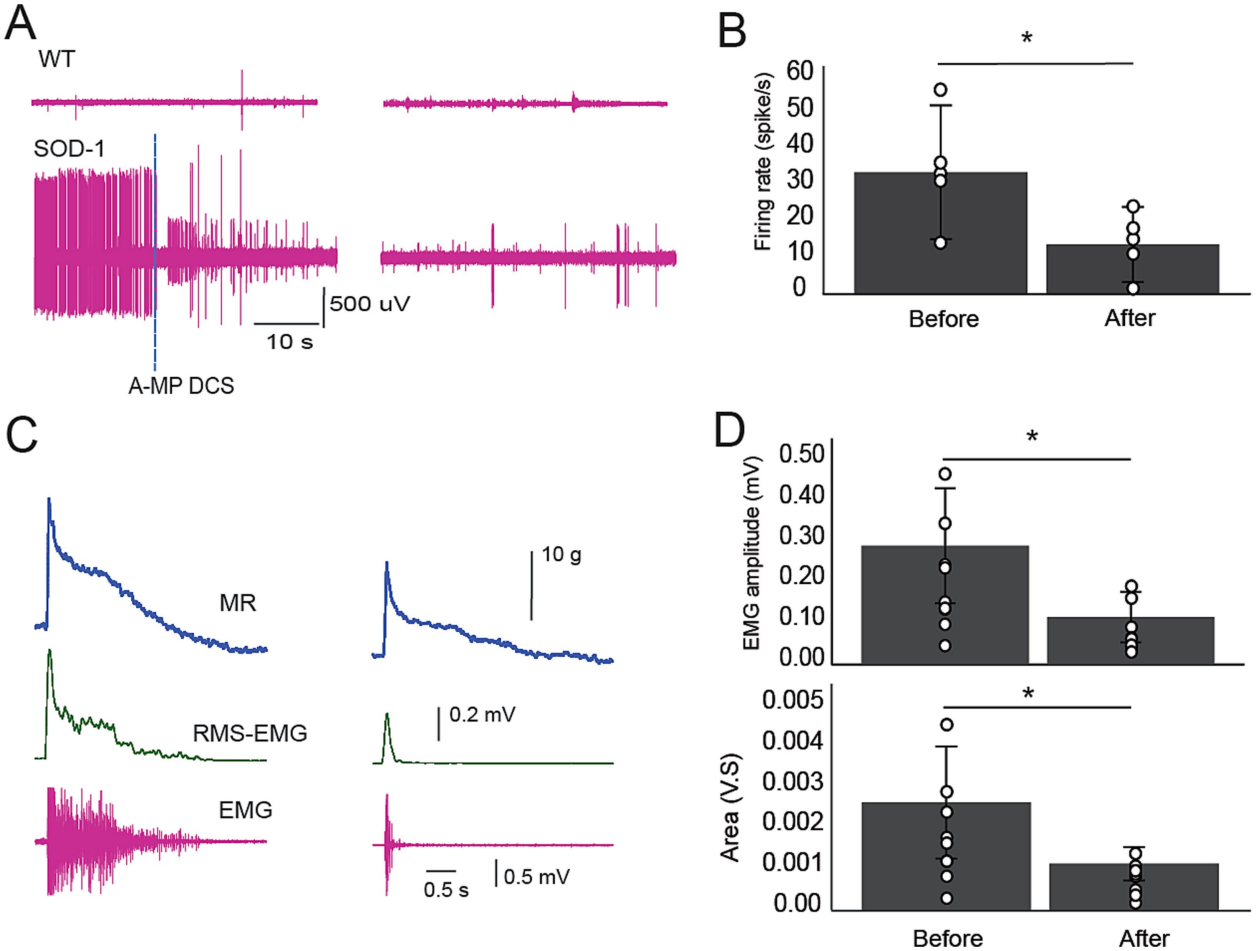
The effects of A-MultiPath-DCS on TA-EMG activity and EMG stretch reflex were measured in the early stage of the disease. The stretch apparatus was used to stretch muscles and concurrently record EMG from the triceps surae muscles. TA-EMG activity was recorded during periods before applying the stretch protocol. **(A)** TA-EMG activity was recorded before and during A-MultiPath-DCS in wild-type (top) and SOD1 (bottom) mice. Note the immediate reduction of TA-EMG activity upon turning on stimulation (A-MPS). **(B)** Firing rate (spikes/s) was calculated and compared before and after A-MultiPath-DCS (*n* = 9; *p* = 0.02). **(C)** Examples of stretch reflexes showing muscle resistance (MR), RMS-EMG, and raw EMG recorded before and after stimulation. **(D)** EMG amplitude and area were significantly reduced after A-MultiPath-DCS (*n* = 9, *p* = 0.02; and *p* = 0.023, respectively, paired t-test).

One of the significant observations we noticed in the SOD1 model is the appearance of TA-EMG activities at the onset of the disease. This TA-EMG activity also emerges in conjunction with tremors of the hindlimbs, especially paws and toes. TA-EMG was episodic in the early stage (about a 2-min episode of activity and about 3-min’ silent intervals). In Figures 3A,B, we show that TA-EMG increases in SOD1 following the onset of the disease and applying A-MultiPath-DCS during the episodes of activity causes immediate reduction of TA-EMG firing rate from 34 ± 6.7 to 13.9 ± 3.7 spikes/s (*n* = 5, *p* = 0.02). Comparably, in later stage, A-MultiPath-DCS causes immediate cessation of TA-EMG activity, and significantly reduces it 1 h after the stoppage of stimulation (*n* = 9, *p* = 0.022), data not shown.

#### Stretch reflex

##### Early-stage

Hyperreflexia is one of the significant symptoms in humans with ALS and in the mSOD1^G93A^ mouse model (9). Hyperreflexia is also linked to the neuropathophysiology of spinal motor neurons (10). The current study induced stretch reflexes using a stretch apparatus (6) to measure muscle resistance and concurrently record EMG responses from the triceps surae muscle. Figure 3C (raw traces) and Figure 3D show that an immediate application of A-MultiPath-DCS significantly reduces the EMG amplitude and area of spinal stretch reflexes (*n* = 9), *p* = 0.02; and *p* = 0.023, respectively, paired t-test.

##### Late-stage

**Figure 4:**
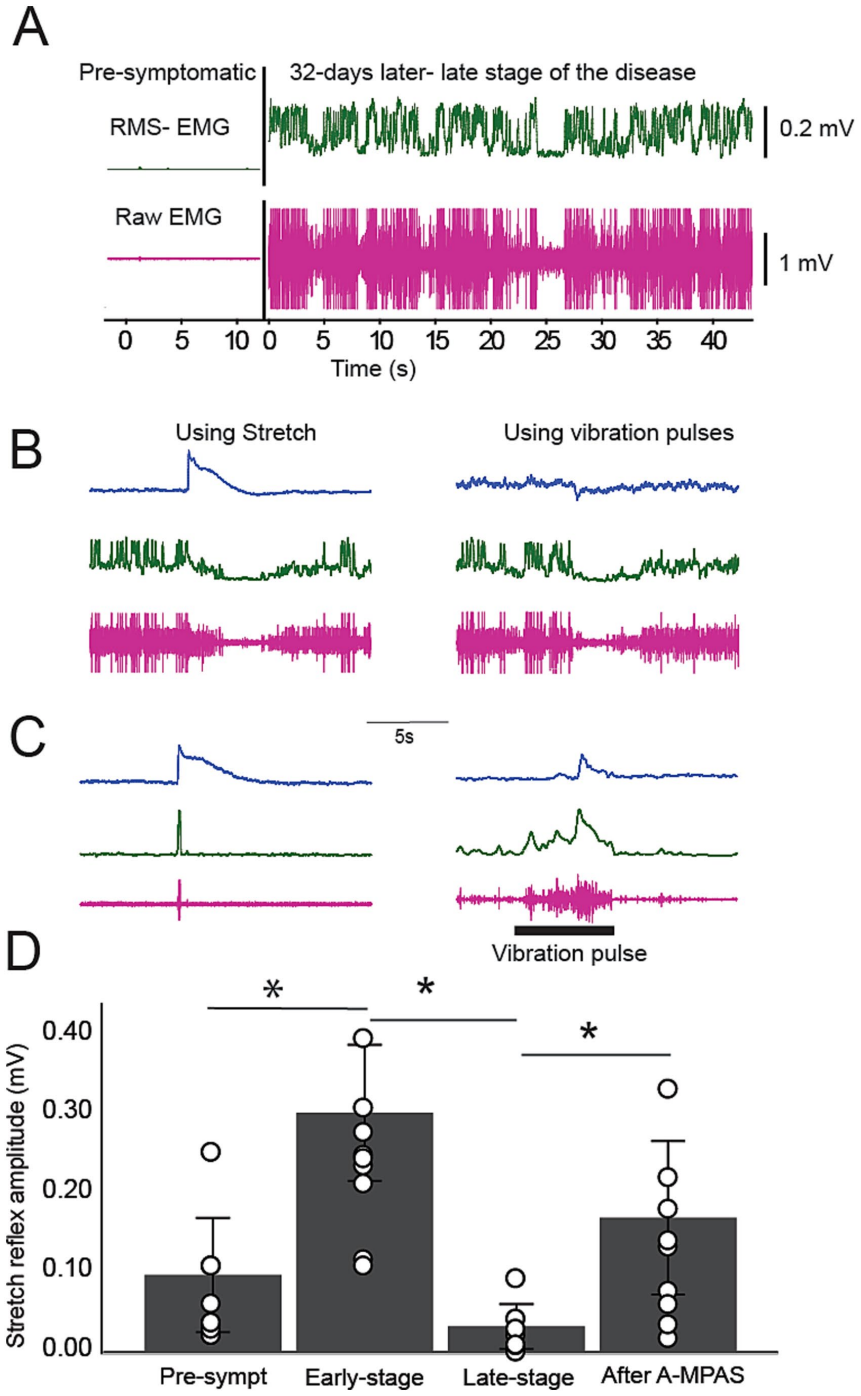
Characterizing TA-EMG activity and its interaction with the stretch reflex responses in later stages of the disease and effects of A-MultiPath-DCS. **(A)** Example of EMG activity recorded from the same animal before onset and 32 days later when the animal was in a late-stage condition. Note that pre-symptomatic TA-EMG was almost absent; however, in the later stage of the disease, TA-EMG was continuous and had a higher amplitude. **(B)** Example of the late-stage stretch reflex and vibration-induced response. Initiating stretch reflex with a stretch device or using a vibration pulse did not induce a reflexive response and caused depression of TA-EMG. **(C)** A-MultiPath-DCS reduced TA-EMG (as shown in Figure 3), which caused the reappearance of the reflexive responses to stretch and vibration. Blue: muscle resistance; green: RMS-EMG; Red: raw EMG. **(D)** Bar graph showing average data from the same group of animals (*n* = 9) at different stages of disease progression and following treatment with A-MultiPath-DCS. At the onset of the disease, animals manifest exaggerated reflex responses significantly higher than pre-symptomatic reflexes (*p* = 0.011, t-test). These exaggerated responses were significantly depressed and mostly not inducible at the late stage of the disease (*p* = 0.027). 60 min of treatment with A-MultiPath-DCS reduces TA-EMG activity and allows stretch reflexes to be expressed, which increased significantly compared to before stimulation (*p* = 0.006).

As mentioned above, we observed that in later stages of the disease, TA-EMG activity becomes continuous and occurs at a higher frequency (Figure 4A), and causes stretch-induced EMG responses to become depressed (Figures 4B–D). Furthermore, muscle stretches cause after-stretch inhibition of TA-EMG activity (Figure 4B). In 9 animals, we measured spinal reflexes in response to stretches (using the stretch apparatus) and vibration pulses. Repeated measures ANOVA shows a significant difference among the time points (*F*_(3,32)_ = 12.06, *p* = 0.0001, Figure 4D). At the onset of the disease, SOD1 animals manifest exaggerated reflex responses significantly higher than pre-symptomatic reflexes (*p* = 0.002). These exaggerated responses were significantly depressed and mostly not inducible at the late stage of the disease (*p* = 0.0001). Following 1 hour of A-MultiPath-DCS treatment, we found there was a reduction of TA-EMG activity which allowed stretch reflexes to return, and increased significantly compared to before stimulation (*p* = 0.023). It is also shown in Figures 4B,C that vibration-induced reflexes are similarly modulated by TA-EMG activity and A-MultiPath-DCS intervention. Together, this data indicates a reversal of reflex responses caused by exaggerated TA-EMG spinal activity, and that A-MultiPath-DCS restores spinal reflexes by reducing this TA-EMG activity.

#### Paw tremor

**Figure 5:**
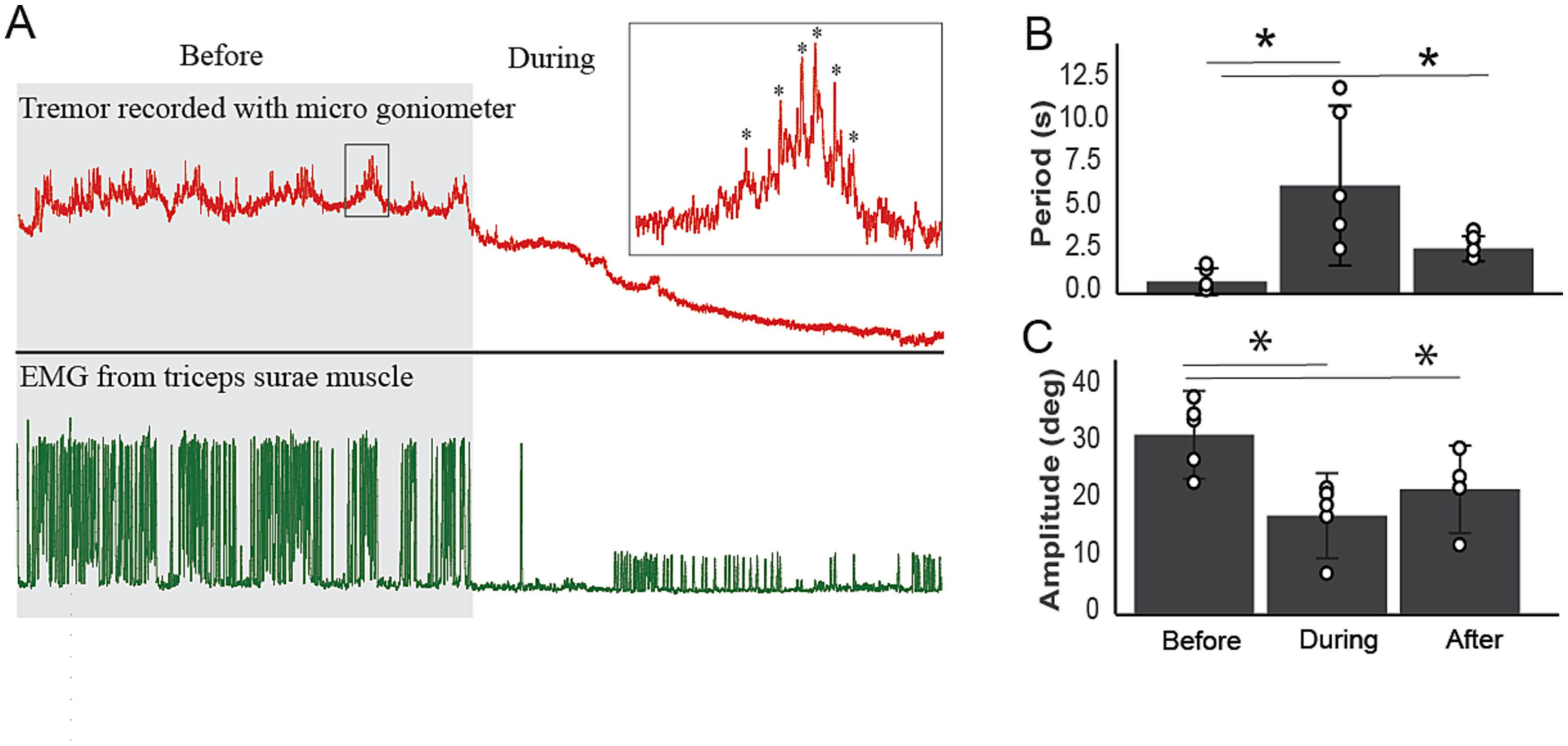
The immediate effects of A-MultiPath-DCS on tremor in the hindlimbs. A microgoniometer recorded tremors to measure the oscillations of the paw. At a range of current intensities (0.5 to 1.5 mA), tremors mostly disappeared. Higher intensities (>1.5 mA) cause the reappearance of tremors and intensify them. Therefore, in 5 animals, we used a current intensity of 1.5 mA to suppress tremors. **(A)** Example of tremor trace and concurrent EMG recorded before and during A-MultiPath-DCS. Note the complete reduction of tremors and the concurrent EMG. **(B)** The period of tremors was significantly increased during and after A-MultiPath-DCS (*F*_(2,12)_ = 7.651, *p* = 0.014, Repeated Measure ANOVA). During stimulation, the period was increased from 0.77 to 6.4 s (*p* = 0.039), and remained high after stimulation ceased (2.7 s, *p* = 0.021). **(C)** The amplitude of tremors was reduced significantly, as shown in panel **(C)** (*F*_(2,12)_ = 7.69, p = 0.014, Repeated measure ANOVA). During stimulation, the tremor amplitude is reduced from 31.4 to 17 uV, (*p* = 0.026). Following the end of stimulation, the amplitude is significantly lower (21.8 uV) compared to before stimulation (*p* = 0.008). This effect was maintained for at least 24 h following 60 min of A-MultiPath-DCS (data not shown). * indicates significance compared to before treatment.

Tremor is one of the leading clinical signs of disease in transgenic mice (11, 12) and humans with ALS (13). To quantify tremors and examine the effect of A-MultiPath-DC, we used a micro goniometer to measure the oscillations of the paw. Figure 5A shows the immediate impact of A-MultiPath-DCS on tremors. At a range of A-MultiPath-DCS intensities (0.5 to 1.5 mA), tremors mostly disappeared. Higher intensities (>1.5 mA) cause the reappearance of tremors and intensify them. Therefore, in 5 animals, we used A-MultiPath-DCS intensity of 1.5 mA to suppress tremors. The period of tremors was significantly increased during and after A-MultiPath-DCS (*F*_(2,12)_ = 7.651, *p* = 0.014, Repeated Measure ANOVA). During stimulation, the period was increased from 0.77 to 6.4 s (*p* = 0.039), and remained high after stimulation ceased (2.7 s, *p* = 0.021) (Figure 5B). The amplitude of tremors was also reduced significantly, as shown in Figure 5C (F_(2,12)_ = 7.69, p = 0.014, Repeated measure ANOVA). During stimulation, the tremor amplitude is reduced from 31.4 to 17 uV, (*p* = 0.026). Following the end of stimulation, the amplitude is significantly lower (21.8 uV) compared to before stimulation (*p* = 0.008) (Figure 5C). This effect was maintained for at least 24 h following 1 h of the A-MultiPath-DCS session.

##### MEP

**Figure 6:**
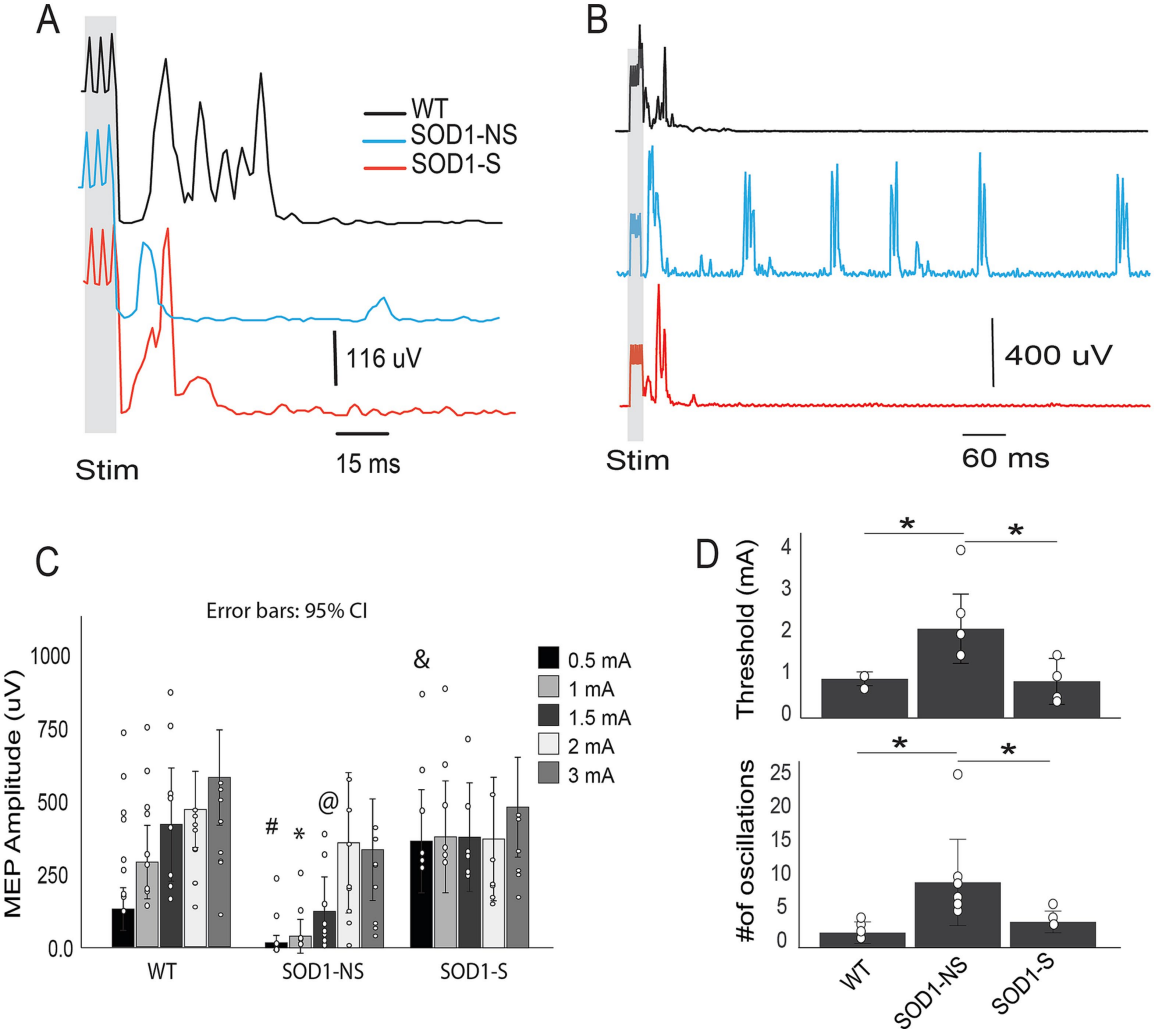
Characterizing motor evoked potential (MEP) in SOD- 1 mice after A- MultiPath- DCS treatment. SOD- 1 mice underwent A- MultiPath- DCS treatment five times over a ten- day period, after which they were anesthetized and tested for motor- evoked potential (wild- type: *n* = 9; SOD 1 non- stimulated: *n* = 8; SOD 1 stimulated: *n* = 6). MEP was elicited by stimulating the hindlimb motor cortical area and recorded from the triceps surae muscles of the contralateral side (to the cortical area). **(A)** Examples of MEPs demonstrating differences in the first responses evoked at 1 mA. **(B)** Examples of MEP showing oscillations following the first responses at a 2 mA cortical stimulus intensity. In this case, oscillations were observed only in the non- stimulated carrier group and persisted for at least 1 min after the cortical stimulus. **(C)** Bar graph showing average MEPs at increasing cortical intensities (0.5 to 3 mA). The MEP amplitude at the lower cortical stimulus intensity of 0. 0.5 mA showed that the SOD 1- nonstimulated (SOD 1- NS) group had significantly lower responses compared to the SOD 1- stimulated (SOD 1- S) group (*p* = 0.000016), but not compared to WT (*p* = 0.119). At the same intensity, the SOD 1- S group also had significantly higher amplitude compared to WT (*p* = 0.001). At 1 mA, SOD 1- NS exhibited significantly lower amplitude than both WT (*p* = 0.006) and SOD- S (*p* = 0.00086). At 1. 1.5 mA, SOD 1- NS recorded significantly lower responses than WT (*p* = 0. 02), but not significantly different from SOD- S (*p* = 0.089). No significant differences were found among the groups at the other intensities tested (2 and 3 mA). **(D)** Top: bar graphs showing the average threshold in mA, and the bottom graph shows the number of oscillations from the 1- pulse stimulation. Comparisons between the groups using ANOVA indicate statistical significance (*F* (2, 14) = 7.67, and *p* = 0.006). The SOD 1- NS group displayed a higher threshold (2.14 ± 0.89 mA, *n* = 7) compared to wild- type (0.94 ± 0.13 mA, *p* = 0.016, *n* = 5) and SOD 1- S (0.88 ± 0.44, *p* = 0.012, *n* = 5), Tukey HSD. Bottom: For the number of post- stimulus oscillations, One- way ANOVA revealed a significant difference among the groups (*F* (2, 12) = 4. 41, *p* = 0.033). The number of post- stimulus oscillations was significantly higher in the SOD 1- NS group for 6- pulse (9.71 ± 6.9) cortical stimulation compared to wild- type (1.5 ± 0.5 and 2.2 ± 1.2, respectively, *p* = 0.039), but not compared to the SOD 1- S group (1.6 ± 0.7 and 3.8 ± 1.3, respectively, *p* = 0.113, Tukey test HSD). # denotes significance between SOD 1- S and SOD 1- NS; & denotes SOD 1- S compared to WT; * denotes SOD 1- NS compared to both SOD 1- S and WT; @ denotes SOD 1- NS significance compared to WT but not SOD 1- S.

To test the integrity of the corticospinal tract and how A-MultiPath-DCS influences it. We analyzed the amplitude, threshold, and number of oscillations of motor-evoked potentials (MEP) (Figure 6).

First, we measured the amplitude of MEP from the muscles (triceps) of hindlimbs of wild-type (*n* = 9), SOD1-NS (*n* = 8), and SOD1-S (*n* = 6) mice. An RM-ANOVA testing shows significant differences among the groups (*F*_(2,20)_ = 7.87, *p* = 0.003). Pairwise comparisons (Bonferroni) shows the following significance: MEP amplitude evoked at lower cortical stimulus intensity of 0.5 mA, the SOD1-NS group showed significantly lower responses compared to the SOD1-S group (*p* = 0.000016) but not to WT (*p* = 0.119). At the same intensity, the SOD1-S was also significantly higher amplitude compared to the WT (*p* = 0.001). At 1 mA intensities, SOD1-NS showed significantly lower amplitude than both WT (*p* = 0.006) and SOD-S (*p* = 0.00086). At 1.5 mA, SOD-NS showed significantly lower responses than WT (*p* = 0.02) but not significantly from SOD-S (*p* = 0.089). There were no significant differences among the groups in other intensities used (2 and 3 mA).

We also calculated the threshold as the EMG response at the lowest cortical train-stimulus intensity possible. Comparison between the groups using ANOVA shows statistical significance (*F*_(2,14)_ = 7.67, and *p* = 0.006). For the 1-pulse stimulation, the SOD1-NS group has a higher threshold (2.14 ± 0.89 mA, *n* = 7) compared to wild-type (0.94 ± 0.13 mA, *p* = 0.016, *n* = 5) and SOD1-S (0.88 ± 0.44, *p* = 0.012, *n* = 5), Tukey HSD. For train stimulation, ANOVA shows significant differences among the groups (*F*_(2,14)_ = 4.12, *p* = 0.039). The SOD1-NS group has a significantly higher threshold (0.97 ± 0.61 mA) compared to the SOD1-S group (0.32 ± 0.14 mA, *p* = 0.041) but is not different from the wild-type (0.5 ± 0.0, *p* = 0.15) Tukey HSD, data is not shown in Figure 6.

For the number of post-stimulus oscillations, One-way ANOVA shows a significant difference among the groups (*F*_(2,12)_ = 4.41, *p* = 0.033). The number of post-stimulus oscillations was significantly higher in the SOD1-NS group for both 1-pulse (3 ± 1.68, data not shown) and 6-pulse (9.71 ± 6.9) cortical stimulation compared to wild-type (1.5 ± 0.5 and 2.2 ± 1.2 respectively, p = 0.039), but not to SOD1-S group (1.6 ± 0.7 and 3.8 ± 1.3), respectively, *p* = 0.113, Tukey test HSD. Data in response to train stimulation is shown in Figures 6A–D; we omitted the 1-pulse data from the figure to clarify and avoid redundancy.

##### Effects of a-multi-path-DCS on HSP70, NKCC1, mSOD1, and spinal motor neuron survival

**Figure 7:**
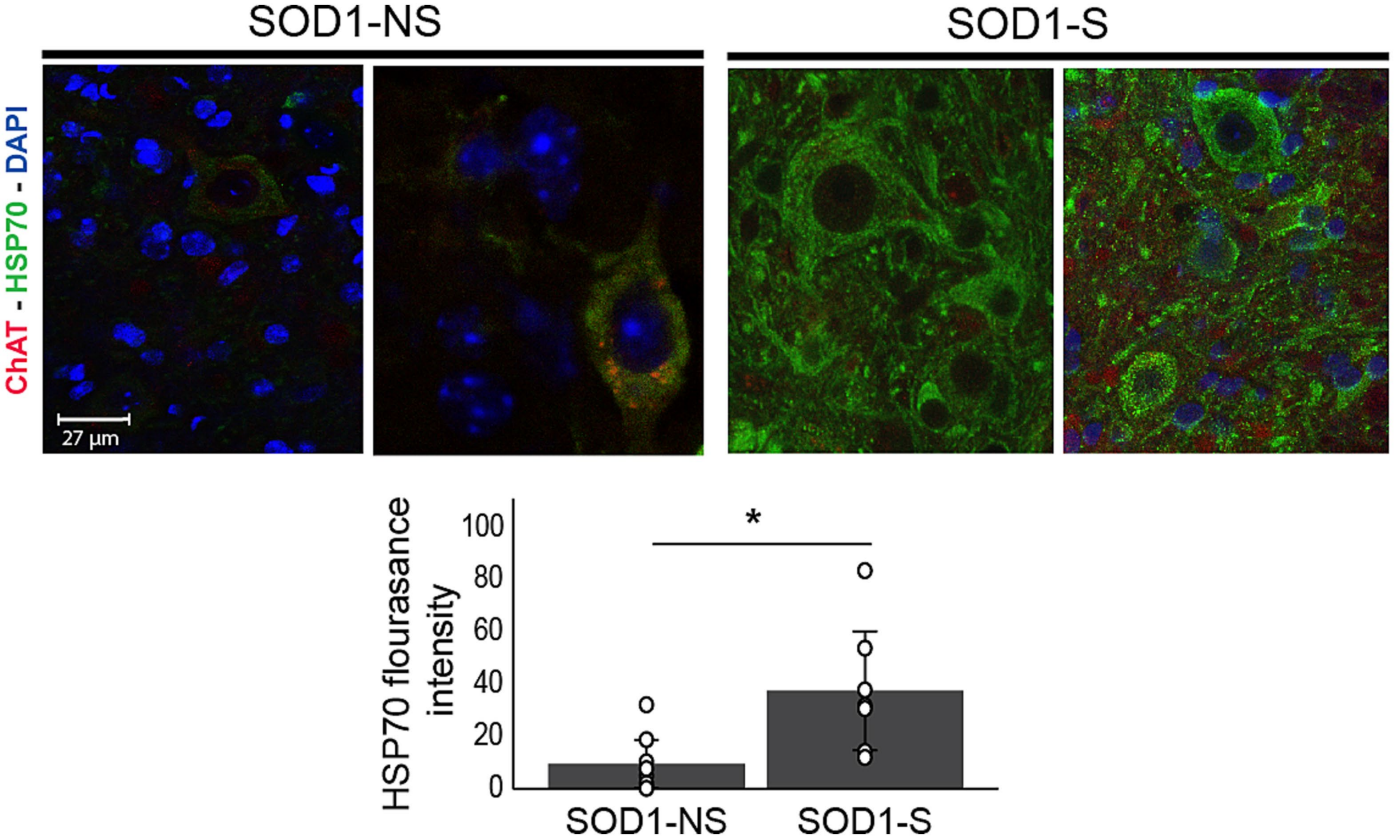
A-MultiPath-DCS upregulates HSP70 expression. HSP70 expression was significantly increased in the spinal cords of stimulated SOD1 mice. The HSP70 immunofluorescence intensity of single spinal motor neurons was calculated using ImageJ. The bar graph represents the average HSP70 intensity in SOD1-Stimulated (SOD1-S) (*n* = 7) and SOD1-nonstimulated (SOD1-NS) (*n* = 8). Images from control wild-type animals are included for reference. *p = 0.012.

Next, we looked at molecular changes that might underlie the observed physiological and functional effects of A-MultiPath-DCS. HSP 70 is neuroprotective and was shown to be upregulated after direct current stimulation (6), and its low level might contribute to motor neuron death. Therefore, we looked at heat shock protein (HSP70) levels in both stimulated (*n* = 7) and nonstimulated animals (*n* = 8) using immunohistochemistry of motor neurons in the lumbar spinal cord. ChAT and DAPI were used to identify motor neurons. As shown in Figure 7, the stimulated group showed significantly higher levels of HSP70 than the non-stimulated group (*p* = 0.012, independent t-test).

**Figure 8:**
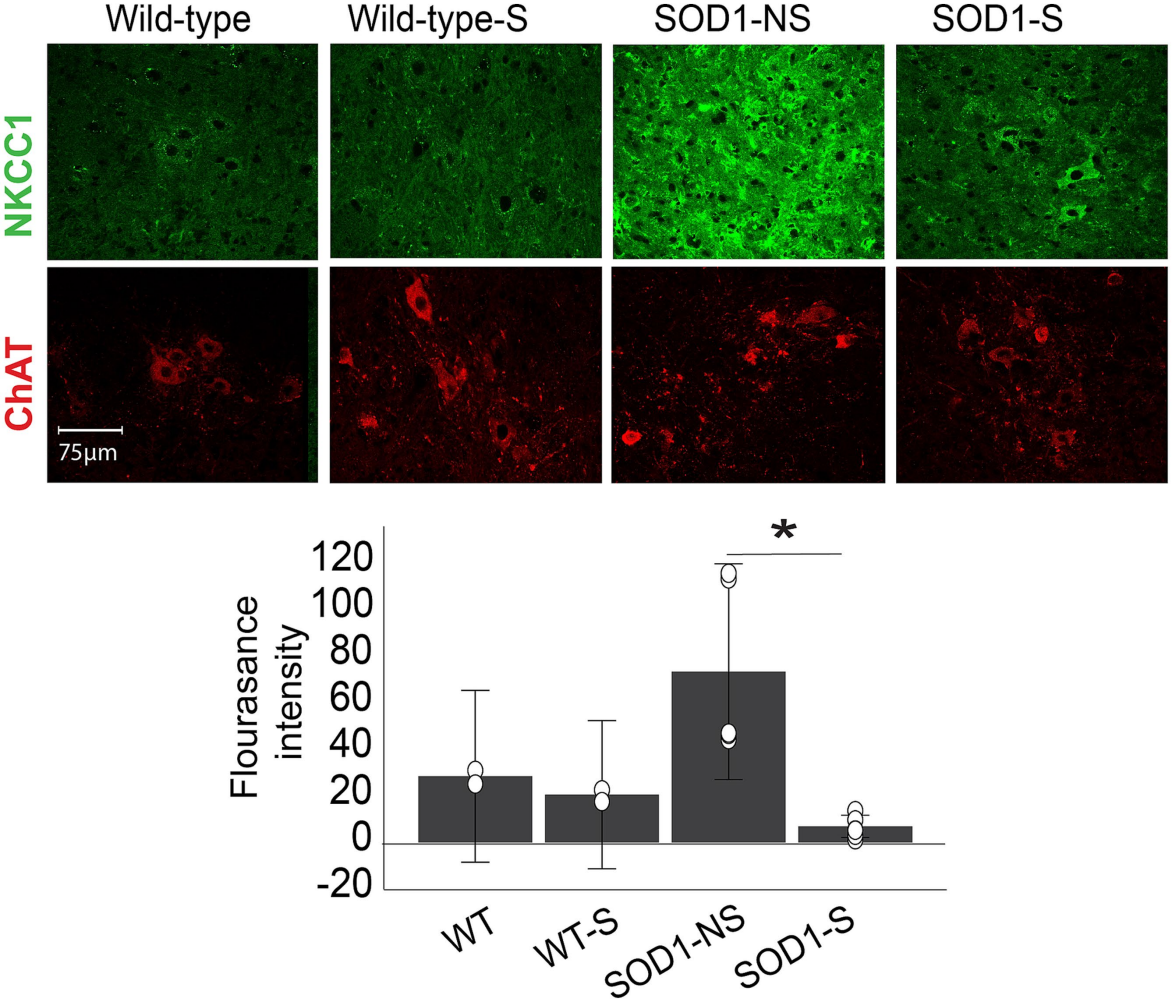
A-MultiPath-DCS reduces NKCC1 expression in spinal motor neurons. Top images: examples of spinal slices from four groups stained for NKCC1 (green). Bottom: bar graph showing average NKCC1 intensities in wild-type non-stimulated (*n* = 2) versus wild-type stimulated (*n* = 2); and SOD-1 non-stimulated (*n* = 6) versus SOD1 stimulated mice (*n* = 5). *p = 0.00009.

NKCC1 is the neuronal chloride transporter that co-regulates GABA and glycine receptors induced inhibition in the spinal cord (14) and could confer the mechanism underlying the excitability suppression by direct current. As shown in Figure 8, the level of NKCC1 protein was measured in motor neurons of stimulated and non-stimulated SOD1 and wild-type animals using IHC. We tested the effects of A-MultiPath-DCS in 4 groups of animals: wild-type stimulated and nonstimulated, SOD1 stimulated and non-stimulated. Independent t-test shows a significantly lower NKCC1 intensity level in the stimulated (*n* = 5) versus the non-stimulated (n = 6) SOD1 groups (*p* = 0.00009). There was also a lower level of NKCC1 intensity in stimulated (*n* = 2) versus non-stimulated (*n* = 2) wild-type animals (not statistically tested, shown for reference).

**Figure 9:**
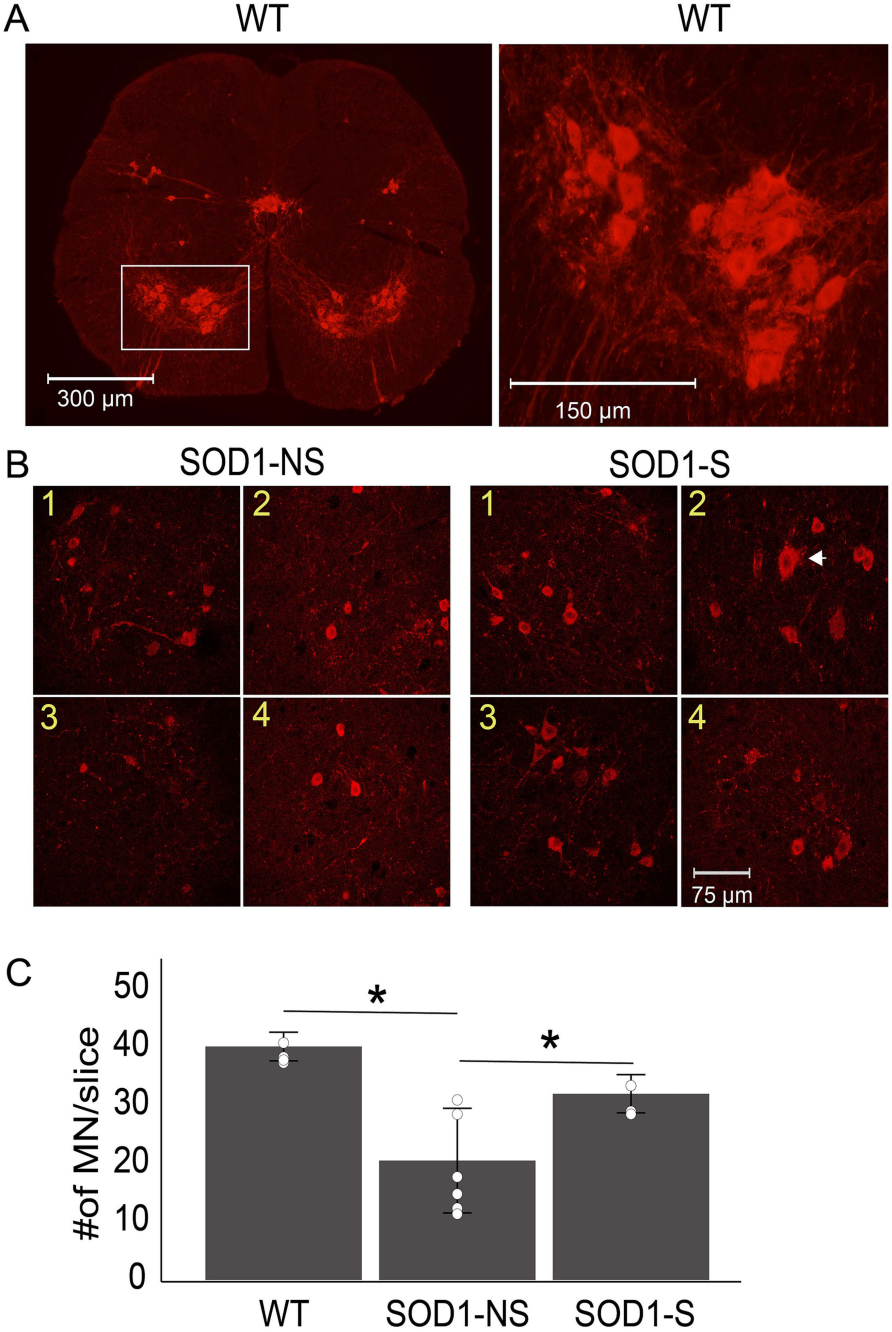
Surviving spinal motor neurons were significantly higher in A-MultiPath-DCS SOD1-stimulated (SOD1-S) animals. Three groups of mice were used to investigate spinal motor neuron survival: Wild-type (*n* = 4), SOD1 non-stimulated (NS, *n* = 6), and SOD1 stimulated (S, *n* = 5). SOD1 mice began real or sham intervention at the onset of the disease. All animals were sacrificed 10 days later. The upper lumbar spinal cord region was sliced (3 slices per mouse) and immunostained for ChAT to mark and count motor neurons. Neurons were counted using the counting functions in Photoshop software. **(A)** Examples of ChAT-stained ventral horns from wild-type animals. This graph shows the entire slice on the left, and the boxed area indicates the region used for counting the ChAT-stained neurons. Cholinergic neurons in the central canal region or dorsal horn were not included in the count. A magnification of the boxed area is displayed on the right side of the graph, which also reveals two main clusters of motor neurons. **(B)** The clusters are shown on the left (1 and 2) and the right (3 and 4) sides of the spinal cord slice for both SOD-NS and SOD1-S. The arrowhead points to a relatively larger neuron that survived in the SOD1-S. **(C)** Bar graph showing the comparison between the three groups. WT has a significantly higher number of spinal motor neurons than the SOD1-NS group (**p = 0.0001) and the SOD1-S group (**p = 0.011); the SOD1-S group has a significantly higher number of neurons than the SOD1-NS group (*p = 0.01).

Spinal motor neurons degenerate in humans with ALS (15) and SOD1 mice (16, 17), and their survival is an important target for any treatment protocol. In Figure 9, spinal motor neurons were counted, and averages were compared among groups. Comparison using one-way ANOVA showed significant differences between groups (*F*_(2,12)_ = 14.4, *p* = 0.0006). As expected, Post-hoc Tukey HSD shows that the WT group has the highest number of motor neurons (*n* = 4, 40.25 ± 1.55) compared to the other two groups (WT to SOD1-NS, *n* = 6, *p* = 0.0005; WT to SOD1-S, *n* = 5, *p* = 0.0006). The SOD1-S group has a significantly higher number of survived motor neurons compared to the SOD1-NS group (32.1 ± 2.66 vs. 20.58 ± 8.5 respectively, *p* = 0.017).

**Figure 10:**
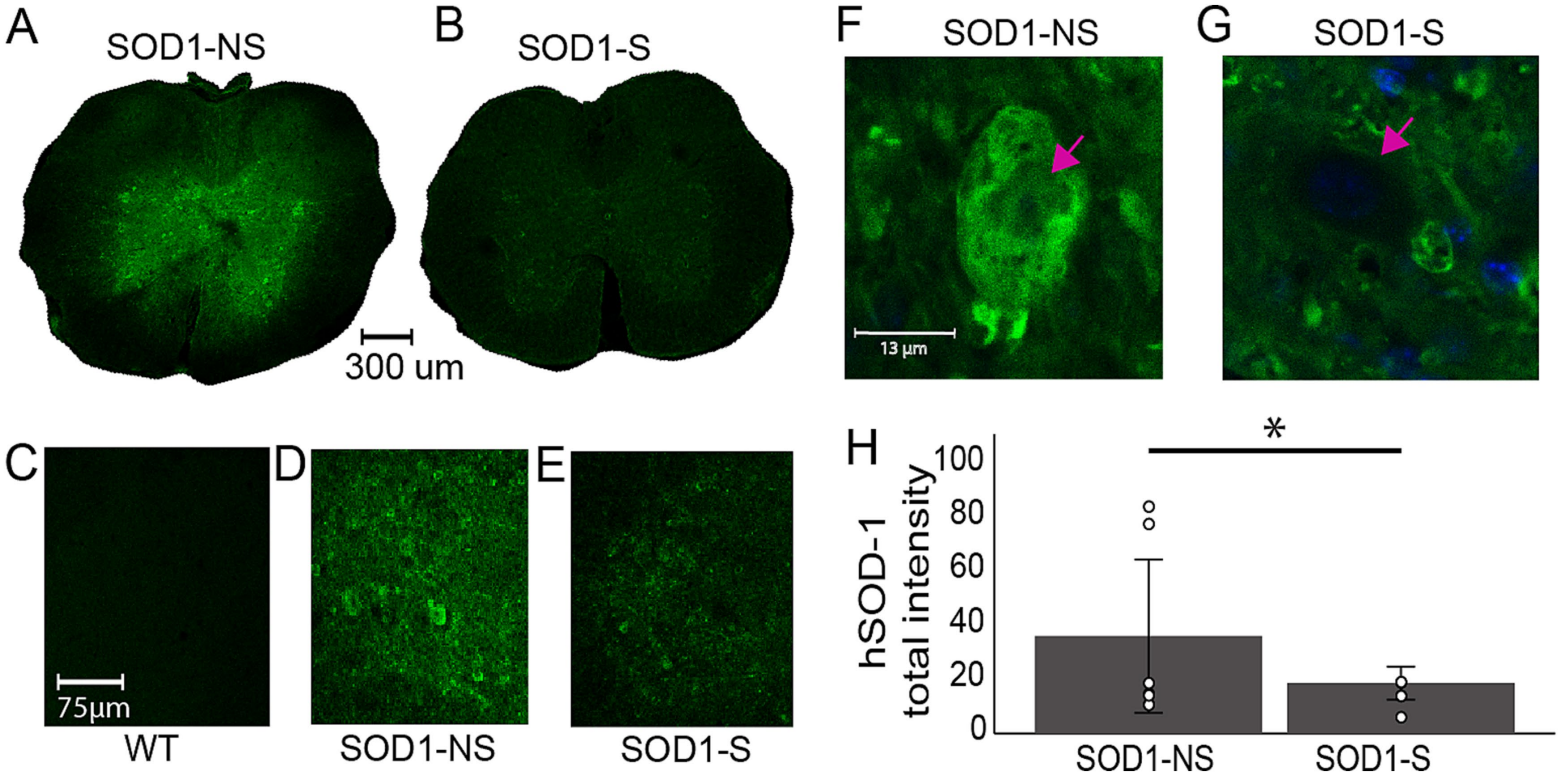
A-MultiPath-DCS reduces the expression of hSOD1. **(A–E)** Photomicrographs of spinal cords in stimulated and non-stimulated mice. **(F)** Bar graph showing significantly lower hSOD-1 intensity in stimulated animals (SOD1-NS: 8 slices from 4 animals; SOD1-S: 5 slices from 5 animals). **(C,D)** Zoom-in confocal images of ventral horns of spinal cords from WT, SOD1-NS, and SOD1-S mice. **(F,G)** Images of single motor neurons showing the level and location of hSOD1 expression. Note that in SOD1-NS, hSOD1 spreads everywhere [cytoplasm and nucleus (arrows)], and that was reduced in SOD1-S. This result was confirmed using z-stack imaging. **(H)** Summary bar graph showing the means of the total intensity of hSOD1 protein in SOD1-NS and SOD1-S mice.

Abnormal human Cu, Zn superoxide dismutase protein (SOD1) in transgenic mice might be affected by the stimulation and confer an underlying mechanism for improved survival in these animals. Therefore, we measured the level of the mutant hSOD1 protein in SOD1-S and SOD1-NS animals. As shown in Figure 10, spinal slices from SOD1-S animals show significantly lower intensity levels of hSOD-1 protein (18.67 ± 4.98) compared to slices from SOD1-NS (36.09 ± 30.79) animals (*p* = 0.005, t-test).

## Discussion

We have developed a novel, non-invasive approach to the modulation of spinal motor neuron activity using multi-site neuromodulation. By using multiple electrodes positioned along the spinal column together with bilateral peripheral nerve electrodes, we can apply uniform current over a larger area of the spinal cord, thereby driving direct current (DC) simultaneously across the spinal cord and down the peripheral nerve in a manner that suppresses motor neuron excitability and induces certain molecular changes that we discuss below. We refer to this approach as “MultiPath-DCS” and “A-MultiPath-DCS” when utilized in the configuration where anode electrodes are positioned along the spinal column. In the present study, we evaluated the effects of A-MultiPath-DCS in the SOD1-G93A mouse model of ALS, which develops motor neuron hyperexcitability during the disease process.

The onset of the disease was determined using the grid walking test, which we have a long experience with (6); however, this is the first study to use in a rodent model of ALS. Therefore, we performed tests to establish its concurrent validity. We used two tests that were used and established in the rodent model of ALS: the wire hang test and the grip strength. Both tests were significantly correlated with the grid walking test scores, as seen in the Supplementary Figure 4 DOI 10.5281/zenodo.13955488.

Using a therapeutic stimulation paradigm (with treatment started following the onset of disease), we report several significant findings: (1) the survival time of SOD1 mice was increased by 74% following treatment with A-MultiPath-DCS. This improvement in survival time was associated with a reduction in the emergence of motor dysfunction, with treated mice retaining motor function better than untreated mice. (2) Both TA-EMG activity and stretch reflex were reduced by A-MultiPath-DCS, indicating that the intervention can modulate these markers of excitability. (3) TA-EMG activity – which is spinal in origin as tested pharmacologically – manifested and progressed predictably in these mice. In the early stages of the disease, TA-EMG was episodic and expressed at a lower frequency. In the later stages, it became continuous and at a higher frequency. The stretch reflex appearance was suppressed by excessive TA-EMG activity. A-MultiPath-DCS reduced TA-EMG and, therefore, allowed the stretch reflex to reappear. This observation could have clinical implications when using reflexes as a marker of excitability in ALS patients. This is important since there are some conflicting findings among studies (18, 19) about the nature of spinal excitability in ALS patients, which – based on the current study, could be due to its relationship with TA-EMG. (4) Tremor in the hindlimbs of SOD1 mice was a very strong component of disease manifestation and was associated with the appearance of TA-EMG. We found that A-MultiPath-DCS could reduce tremor significantly, and in some cases completely, and this effect lasted for at least 24 h. This finding is also in line with the results showing a reduction of TA-EMG. (5) Another abnormality in spinal excitability that was observed in the present study is the repeated oscillations following MEP in SOD1 control mice. This was also associated with a higher MEP threshold that could be an adaptation for spinal hyperexcitability. A-MultiPath-DCS reduced oscillations and MEP threshold in treated mice. This could be one of the factors that led to motor function improvement in the stimulated SOD1 mice. In addition, the MEP findings show the complex effect of A-MultiPath-DCS on the corticospinal excitability in animals with SOD-1 mutation. For example, the higher threshold in the SOD1-NS group probably indicates the dysfunctional connectivity in the corticospinal tract due to synaptic abnormality or failure (20, 21), and the obvious increase in the number of oscillations in the SOD1-NS group indicates the loss of spinal inhibition (22) that was preserved in the SOD1-S group. (6) Regarding molecular changes in response to A-MultiPath-DCS, there was a common theme of downregulating abnormal proteins: hSOD1, NKCC1, and Tau, see Supplementary data at: DOI 10.5281/zenodo.13955488. This was associated with the upregulation of HSP70 in a similar way to previous findings (6). In addition to our previous published results, this data indicates the involvement of common protein clearance mechanisms such as autophagy and proteasomal pathways. (7) Spinal motor neurons were found to be protected from degeneration by A-MultiPath-DCS. This might result from two stimulation-related effects: (1) increased efficiency of protein clearance in these neurons and (2) reduced spinal hyperexcitability. It is well established that direct current stimulation modulates brain and spinal cord excitability in a predictable manner that depends on the polarity of the current facing the targeted tissue. Cathodal stimulation increases excitability, while anodal stimulation reduces excitability in the spinal cord (8, 23–26).

There is always the possibility that the current could spread rostrally, as shown in modeling studies (27), potentially affecting the phrenic nerve and related nuclei, thus improving survivability. However, we did not investigate this because it was not our goal. Another point is that the endpoint in this study was not death; instead, it was when the animal could not right itself from a supine position in less than 20 s—see Table 1. These animals were still able to breathe effectively when they were sacrificed.

The effects of spinal DC stimulation on spinal cord neurons were recently studied. Most studies found that tsDCS can modulate spinal motor neurons in wild-type and SOD1 mice. For example, a study by Jankowiak and colleagues showed that 15 min of anodal or cathodal tsDCS differentially altered EPSP, afferent activity, and membrane passive properties in the SOD1 mice (28). Similarly, Bączyk and colleagues showed differential effects of both anodal and cathodal tsDCS on spinal motor neuron firing properties (29). A detailed discussion on this topic was presented in recent reviews (30, 31). One study tested the application of tsDCS on awake SOD1 mice survival and motor function and showed a different outcome between anodal and cathodal tsDCS (32). Anodal showed a trend of shorter survival but no effects on the progression of motor function decline, and cathodal had no impact. A few differences between our study and the above study might explain the different outcomes: (1) our study uses a different stimulation paradigm as described in the method section, where current is distributed among six electrodes and three current sources. As explained before, that was designed to help direct the current toward the spinal cord and maintain uniformity of the current among the spinal electrodes. (2) The intensity and duration of the applied current were different. (3) the restraining system was also different, which might cause stimulation to be misdirected or interrupted. (4) The frequency of stimulation sessions per week was also different daily versus three times per week. (5) The mice line might have something to do with animals’ response to treatment. (6) There were also behavioral assessment differences. This clearly shows that the effects of different variables of DC application are currently unknown, and further comparable studies of such variables are needed.

**Figure 11:**
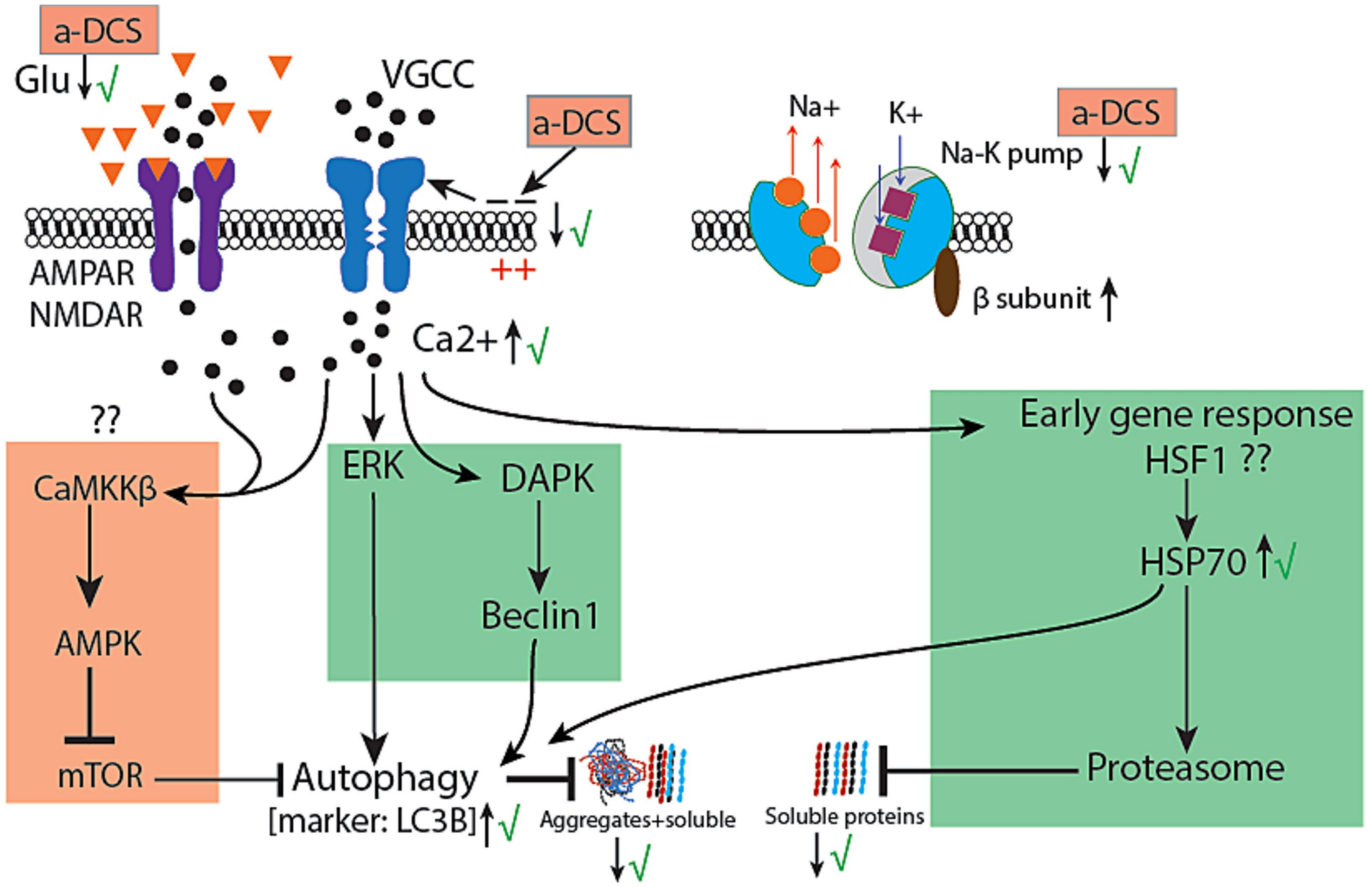
Summary diagram of the proposed mechanism of action. The diagram summarizes the published data on the effects of direct current on cellular mechanisms such as AMPA and NMDA receptors, voltage-gated calcium channels (VGCC), and the sodium-potassium pump, along with their potential downstream cellular effects. The check mark indicates that the results are based on published or unpublished data. The question mark signifies a hypothetical pathway that has not yet been experimentally tested. The conclusion is that anodal direct current stimulation activates a universal protein clearance mechanism applicable to many misfolded proteins. Black dot: calcium molecule; Orange triangle: Glutamate; a-DCS: anodal DCS; VGCC: voltage-gated calcium channel; HSF1: heat shock factor −1. See text for further explanation.

The present results show spinal hyperexcitability to be remarkably consistent and associated with the commencement and progression of motor dysfunction in the SOD1 mice. The most significant mode of hyperexcitability was the excessive TA-EMG activity that has an obvious spinal origin. Nasal application of isoflurane completely stopped intramuscular recorded TA-EMG activity, which is a strong indication that it originated from the spinal cord since isoflurane does not have a significant direct effect on muscle-originated activity (33) but has a strong inhibitory effect on spinal motor neurons (34, 35). The source of hyperexcitability in motor neurons seems to be brought about by intrinsic changes in motor neurons, such as changes in the axonal potassium channel expression (36), morphology (37) and intrinsic membrane hyperexcitability (38). There are also recent findings indicating dysfunction of the spinal inhibitory interneurons in SOD-1^G93A^ mice (39). Excessive TA-EMG spinally originated EMG activity that we have seen in our data could be partially explained by a reduced inhibitory spinal pathway (14, 40, 41). One substantial new piece of evidence from our study (Figure 8) is the significant increase in the level of NKCC1 in the SOD1 mouse spinal cord, which has an impact on cytosolic chloride concentration. This has similarities to post-ischemic injuries or epilepsies in which stresses on neurons lead them to express high levels of NKCC1 and down-regulate KCC2 (42). In this paper, the underlying mechanism was shown to be increased activity of NMDA and MGluR, which leads to an increase in cytosolic Ca + level. This same mechanism operates in ALS, in which both extracellular glutamate and cytosolic Ca + levels are chronically elevated (43, 44). Additionally, plasmalemmal Na+/K + -ATPase is shown to be dysfunctional in ALS (45), which increases the level of extracellular potassium gradients, which in turn increases the activity of NKCC1 (46). Dysfunctional Na+/K + -ATPase and increased level and activity of NKCC1 would cause intrinsic hyperexcitability of motor neurons and disinhibition due to reduced effectiveness of inhibitory spinal interneurons. A-MultiPath-DCS could regulate many of the above hyperexcitability-causing factors (Figure 11).

The effects of applied spinal DCS on neurons have been well studied. First, spinal DCS causes subthreshold changes in the membrane potential of motor neurons, mimicking neuronal activity. In response to dorsal-to-ventral applied spinal DCS (with the anode electrode placed on the dorsal aspect of the spinal cord), Eccles et al. (54) demonstrated, using intra- and extracellular recordings, a pattern of changes in membrane potential in different parts of primary afferent axons (pre-synaptic) and the cell body and initial axon segment of spinal motor neurons (54). These changes in membrane potentials have been replicated in several studies (12, 14, 55). There are immediate neurophysiological effects of membrane potential polarization, such as modifying the firing properties of motor neurons and presynaptic neurotransmitter release (39, 54). For example, anodal-DCS (dorsal) increases the frequency of miniature EPSPs but decreases evoked EPSPs (54–56). Beyond these physiological effects, changes in membrane potential (depolarization) act as a potent signal that activates distinct programs of early response genes (c-fos and c-jun) (57, 58) and regulates growth factor gene expression (59). These responses are induced by calcium entry into the cytoplasm. Stimulating synaptic activity has been shown to exert neuroprotective effects by enhancing autophagy and lysosomal activity (60, 61) and clearing toxic misfolded proteins (62). The second known effect of direct current stimulation on cellular structures is electrophoresis or electro-osmosis of membrane proteins, which could occur in two ways: (1) most integral membrane proteins are negatively charged and therefore interact with the applied field; and (2) charged K + and Na + ions also interact with the applied field and move toward the cathodal direction. These two factors eventually result in the movement of membrane proteins toward the cathode. Although there is no direct evidence that electrophoresis or electro-osmosis operates at the whole animal level, strong *in vitro* evidence shows that the physiological states of the electrical field cause movements in cell membrane proteins within 1 to 2 min. This action could cause, for example, receptor asymmetry, which might significantly affect the neuronal response to environmental signals like glutamate or dopamine molecules, thus modulating neuronal excitability. DCS has been shown to flow in extracellular spaces and cause plasmalemmal membrane potential changes that may mediate its main effects through calcium-dependent mechanisms. Calcium can trigger numerous cellular activities. It should be noted that the DCS-induced increase in cytosolic calcium level is transitory, which differs from the disease-induced increase in cytosolic calcium that is chronically elevated in ALS. Another factor to consider—although no direct evidence supports it yet—is that since about 10% of DC flows intracellularly, it might affect internal organelles like mitochondria (implicated in ALS). Mitochondria have a membrane potential that could interact with applied fields. Additionally, mitochondrial function is sensitive to the ionic composition of the mitochondrial milieu, which is affected by applied electrical currents (63).

Anodal direct current stimulation showed a neuroprotective effect in a rat ischemic-stroke model (47, 48). Spinal and cranial direct current stimulation increased BDNF (49–51). The expression of GDF5 (growth/differentiation factor 5) and PDGFA (platelet-derived growth factor subunit A) were increased following direct current stimulation (48). Moreover, VEGF expression increased following spinal DC (Ahmed, unpublished) and cranial DC (48). Enhancement of these growth factors could be an underlying mechanism protecting motor neurons in SOD1 mice. We should note that in the current study, the type of motor neurons was not identified in terms of fast versus slow. Therefore, we do not know which type has survived due to the stimulation.

DC stimulation of neural tissues was also found to cause a transient increase in cytosolic calcium levels (52). This transient increase in cytosolic calcium activates multiple calcium-dependent processes. One of these processes enhances the protein degradation pathway and blocks apoptosis. In Figure 11, we summarize the different potential mechanisms by which DC stimulation could enhance protein mechanisms such as autophagy and the proteasomal systems. One piece of supportive evidence is the autophagy marker, LCB3, which was increased acutely following stimulation; see Supplementary Figure 3, DOI 10.5281/zenodo.13955488.

Glutamate-induced cytotoxicity has been shown to play a role in ALS pathology. In ALS patients, plasma levels of glutamate are increased. Surprisingly, soluble factors (SOD1-SF)1-SF.)1-SF) collected from patients with ALS caused excitotoxicity in neuronal cultures. There is overwhelming evidence showing that high levels of extracellular glutamate accumulate in the CNS of ALS patients. Our current data show high neuronal activity of spinal motor neurons in SOD-1 G93A mice. This continuous spinal activity could be the source of the elevated glutamate levels in the CNS. Chemically induced membrane potential depolarization has been shown to protect against glutamate-induced cytotoxicity. This protection is suggested to arise from depolarization-induced inactivation of voltage-gated calcium channels (VGCCs), which reduces cytosolic calcium that triggers cell death mechanisms. Repeated direct current stimulation of the spinal cord leads to an adaptive response in motor neurons manifested as a depolarization of membrane potential. This DC-induced long-term adaptive response provides another layer of protection against cytotoxicity in ALS. Additionally, evidence from our lab and others shows reduced spinal excitability in response to repeated anodal spinal DCS, which is mediated by enhanced spinal inhibition. This DC-induced inhibition could represent another mechanism protecting motor neurons from overload activity.

Our previous study found that anodal spinal DCS causes significant upregulation of HSP70 (6) in a spinal cord injury model. This study also shows a direct relationship between degradation pathway activation, HSP70 expression, and DCS. Moreover, unpublished data from *in vitro* experiments have confirmed the *in vivo* results (6). It has been reported that HSP70 is involved in proteasomal degradation of mutant SOD1 (53). Since our current studies reveal that anodal DCS induces upregulation of HSP70 in SOD1G93A mice, we believe this action of A-MultiPath-DCS to be a significant factor contributing to slowing down motor neuron death.

### Clinical implications

ALS is a rapidly progressive neurodegenerative disease that affects both upper and lower motor neurons. So far, there is no cure or effective treatment for ALS, despite the availability of five FDA-approved therapies to date. SOD1-associated ALS results from mutations in the antioxidant enzyme Zn/Cu ion-binding superoxide dismutase 1 (SOD1), which promote the misfolding and aggregation of the protein in motor neurons. Generally, in ALS, motor neurons appear to be subjected to stressful factors (genetic or environmental) that disrupt protein homeostasis, leading to subsequent cellular disruptions that ultimately result in cell death. In the current study, we investigated the therapeutic effects and possible mechanisms of a novel form of non-invasive multi-site neuromodulation that activates or enhances a common mechanism, increasing the capacity of motor neurons to withstand disease-promoting factors. Multi-site neuromodulation utilizing spinal DCS has recently been shown to be clinically effective in modulating spinal cord excitability in humans with another disorder related to motor neuron hyperexcitability (post-stroke spasticity). In ALS, the premise of suppressing motor neuron hyperexcitability as a potential therapeutic strategy has been supported by recent clinical trials of retigabine, a KCNQ channel activator, and mexiletine, a sodium channel blocker. The approach we describe for implementing non-invasive multi-site neuromodulation is fully scalable for human clinical use in treating ALS and other motor neuron diseases. Furthermore, we expect this approach to synergize with existing and developing pharmacologic and nucleic acid-based therapies for ALS.

## Conclusion

The current study provides crucial data that establishes for the first time the beneficial therapeutic effects of A-MultiPath-DCS in the SOD1-G93A mouse model of ALS and offers fundamental electrophysiological, cellular, and molecular characterization of the treatment effects. A-MultiPath-DCS is a novel form of multi-site neuromodulation that delivers spinal direct current stimulation through multiple electrodes to optimize electrical current uniformity and directionality in the target tissues. A-MultiPath-DCS caused an immediate reduction in spinal cord excitability, measured using TA-EMG, stretch reflexes, tremors, and MEP. Repeated treatments with A-MultiPath-DCS were associated with a decrease in the rate of motor dysfunction emergence and extension of the lifespan of treated SOD1 mice. Repeated treatments also increased the survival of spinal motor neurons and simultaneously upregulated essential proteins like HSP70, which is a known proteasome and chaperone-assisted selective autophagy (CASA) associated protein (71). This process enhanced the clearance of toxic proteins such as hSOD1, phosphorylated Tau, and the hyperexcitability-associated protein NKCC1. The direction of the effect on autophagy may be therapeutically beneficial, as inhibiting autophagy has been shown to accelerate the degeneration of spinal motor neurons in SOD1 mice (72). In conclusion, A-MultiPath-DCS exerts its therapeutic effects in this ALS model by reducing hyperexcitability and enhancing mechanisms for clearing toxic proteins. In conclusion, A-MultiPath-DCS exerts its therapeutic effects in this ALS model by reducing hyperexcitability and enhancing toxic protein clearance mechanisms.

## Data Availability

The raw data supporting the conclusions of this article will be made available by the authors, without undue reservation.
